# High-Throughput Chemical Screen Identifies a Novel Potent Modulator of Cellular Circadian Rhythms and Reveals CKIα as a Clock Regulatory Kinase

**DOI:** 10.1371/journal.pbio.1000559

**Published:** 2010-12-14

**Authors:** Tsuyoshi Hirota, Jae Wook Lee, Warren G. Lewis, Eric E. Zhang, Ghislain Breton, Xianzhong Liu, Michael Garcia, Eric C. Peters, Jean-Pierre Etchegaray, David Traver, Peter G. Schultz, Steve A. Kay

**Affiliations:** 1 Division of Biological Sciences, University of California San Diego, La Jolla, California, United States of America; 2 Genomics Institute of the Novartis Research Foundation, San Diego, California, United States of America; 3 Department of Chemistry, The Scripps Research Institute, La Jolla, California, United States of America; 4 Department of Neurobiology, University of Massachusetts Medical School, Worcester, Massachusetts, United States of America; Howard Hughes Medical Institute/Stanford University, United States of America

## Abstract

The circadian clock underlies daily rhythms of diverse physiological processes, and alterations in clock function have been linked to numerous pathologies. To apply chemical biology methods to modulate and dissect the clock mechanism with new chemical probes, we performed a circadian screen of ∼120,000 uncharacterized compounds on human cells containing a circadian reporter. The analysis identified a small molecule that potently lengthens the circadian period in a dose-dependent manner. Subsequent analysis showed that the compound also lengthened the period in a variety of cells from different tissues including the mouse suprachiasmatic nucleus, the central clock controlling behavioral rhythms. Based on the prominent period lengthening effect, we named the compound longdaysin. Longdaysin was amenable for chemical modification to perform affinity chromatography coupled with mass spectrometry analysis to identify target proteins. Combined with siRNA-mediated gene knockdown, we identified the protein kinases CKIδ, CKIα, and ERK2 as targets of longdaysin responsible for the observed effect on circadian period. Although individual knockdown of CKIδ, CKIα, and ERK2 had small period effects, their combinatorial knockdown dramatically lengthened the period similar to longdaysin treatment. We characterized the role of CKIα in the clock mechanism and found that CKIα-mediated phosphorylation stimulated degradation of a clock protein PER1, similar to the function of CKIδ. Longdaysin treatment inhibited PER1 degradation, providing insight into the mechanism of longdaysin-dependent period lengthening. Using larval zebrafish, we further demonstrated that longdaysin drastically lengthened circadian period in vivo. Taken together, the chemical biology approach not only revealed CKIα as a clock regulatory kinase but also identified a multiple kinase network conferring robustness to the clock. Longdaysin provides novel possibilities in manipulating clock function due to its ability to simultaneously inhibit several key components of this conserved network across species.

## Abstract 2

A novel compound “longdaysin” was found to dramatically slow down the speed of the circadian clock through simultaneous inhibition of protein kinases CKIδ, CKIα, and ERK2.

## Author Summary

Most organisms show daily rhythms in physiology, behavior, and metabolism, which may be advantageous because they anticipate environmental changes thus optimize energy metabolism. These rhythms are controlled by the circadian clock, which produces cyclic expression of thousands of output genes. More than a dozen components of the circadian clock are called clock genes, and the proteins they encode form a transcription factor network that generates rhythmic gene expression. In this study, we set out to control the function of the circadian clock and to identify new clock proteins by means of chemical tools. We tested the effects on the clock in human cells of around 120,000 uncharacterized compounds. Here we describe identification of a novel compound “longdaysin” that markedly slows the circadian clock both in cultured mammalian cells and in living zebrafish. By using longdaysin as a chemical probe, we found new proteins that modulate clock function. Because defects of clock function have been linked to numerous diseases, longdaysin may form the basis for therapeutic strategies directed towards circadian rhythm-related disorders, shift-work fatigue, and jet lag.

## Introduction

A variety of physiological processes such as sleep/wake behavior, body temperature, hormone secretion, and metabolism show daily rhythms under the control of the circadian clock which is intrinsic to the organism. Perturbation of clock function has been implicated in numerous pathologies including circadian sleep disorders, cardiovascular disease, cancer, and metabolic disease [1]–[4]. The close association of the circadian clock with diverse physiological processes and diseases implies that identification of clock-modulating compounds could form the basis for therapeutic strategies directed towards circadian rhythm-related disorders, shift-work fatigue, and jet lag.

The manifestation of circadian disorders at the level of the whole organism can be caused by dysfunction of the clock at the level of intracellular networks, as single cells exhibit circadian rhythms in a cell-autonomous manner [5]–[6]. In mammals, these cellular oscillators are organized in a hierarchy, in which the suprachiasmatic nucleus (SCN) of the hypothalamus constitutes the central circadian pacemaker controlling behavioral rhythms, while peripheral clocks in other tissues control local rhythmic outputs [1],[3],[7]. In the intracellular circadian network, the clock genes and their protein products form transcriptional feedback loops: CLOCK and BMAL1 transcription factors activate expression of *Per* and *Cry* genes, and PER and CRY proteins (PER1, PER2, CRY1, and CRY2) in turn inhibit their own transcription to generate rhythmic gene expression [3],[8].

In addition to transcriptional regulation, post-translational modification of clock proteins provides another level of regulation, as most clock proteins undergo rhythmic phosphorylation [9]. Hamster *tau* mutants showing a short period behavioral rhythm have a missense mutation in the *CKIε* gene [10], and human familial advanced sleep phase syndrome (FASPS) with early sleep times is attributed to missense mutations of *PER2* and *CKIδ* genes [11]–[12]. CKIδ and CKIε phosphorylate PER to trigger proteasomal degradation, and *tau* and FASPS mutations lead to higher PER degradation than wild type, consistent with the short period phenotype [13]–[15]. Supporting the functional importance of CKIδ/ε, application of the known CKI inhibitors IC261, CKI-7, and D4476 causes period lengthening in cultured cells [14],[16]–[17]. Generation of CKIε and CKIδ deficient mice [15],[18] as well as the development of the CKIε-selective inhibitor PF-4800567 [19] revealed the minimal, if any, role of CKIε in period length regulation and also demonstrated a dominant role for CKIδ. In contrast, potential roles of CKI family members other than CKIδ/ε are less characterized: They show much less binding with PER1 than that of CKIε [20]–[22], and knockdown of CKIα-like, a homolog of CKIα, has no period effect in cultured cells [23]. Together with CKIδ/ε, GSK-3β and CK2 are also implicated in period regulation. GSK-3β phosphorylates PER2, CRY2, REV-ERBα, CLOCK, and BMAL1 for functional regulation [24]–[28], and pharmacological and RNAi-based inhibition of GSK-3β causes period shortening in cultured cells [29]–[30]. Conversely, inhibition of CK2 causes period lengthening [29],[31]–[33], and CK2-mediated phosphorylation regulates PER2 and BMAL1 functions [31]–[32],[34].

Genetic and molecular biological studies over the past two decades have identified more than a dozen genes that form the core of the mammalian circadian network [3],[8],[35]. However, it is clear that more clock components and modulators remain to be discovered [36]. Considering the limitations of conventional biological approaches associated with lethality, pleiotropy, and functional redundancy of closely related proteins, introduction of new strategies will accelerate the identification of novel clock mechanism. Chemical biology approaches are attractive candidates, because they utilize small molecules as proof-of-concept probes for biological systems and can be effective in discovering novel biological mechanisms and evaluating their effects in vivo by complementing the limitations of conventional biological approaches [7],[37]. Furthermore, the circadian clock network can be a good target for chemical biology approaches due to the quantitative readout of an oscillation. To discover new chemical probes for dissecting biological mechanisms, it is valuable to screen comprehensive, large-scale compound libraries containing hundreds of thousands of compounds, because a wide variation of chemical structures has the advantage of probing many classes of potential targets. Although it is technically challenging to identify proteins specifically affected by a novel compound [38], this process might also be necessary for known compounds, given that even well-characterized kinase inhibitors have off-targets unrelated to the primary effect [39]–[40]. Combined with conventional biological approaches, the chemical biology approach is expected to provide an effective way to identify novel components of the circadian clock [41].

We previously developed a cell-based high-throughput circadian assay system to perform compound screening [29]. In this system, *Bmal1-dLuc* reporter cells derived from human U2OS osteosarcoma cells show robust luminescence rhythms on 384-well plates by expressing a rapidly degradable luciferase under the control of a mouse *Bmal1* gene promoter. We initially tested a chemical library containing 1,280 well-characterized compounds (LOPAC; Library of Pharmacologically Active Compounds) and found 11 compounds that change period length of the luminescence rhythms in a dose-dependent manner. The kinase inhibitors among the hit compounds revealed novel roles of GSK-3β and CK2 in the mammalian clock mechanism as described above. Furthermore, many of the hits were previously known to alter the circadian period in other organisms and tissue preparations, demonstrating the predictive value of the high-throughput assay system [29]. Together, these observations indicate the effectiveness of small molecules as probes and/or modulators of the circadian clock mechanism. A similar LOPAC screen in NIH3T3 and U2OS cells identified CKIδ/ε-dependent phosphorylation as a chemically sensitive process of the clock [42]. It was found that the CKIδ/ε-targeting compounds cause much larger period lengthening than CKIδ gene knockout [18],[29],[42], but the molecular mechanism underlying the strong effects of the compounds remains unknown. The present study aimed to apply chemical biology methods to probe the clock mechanism with novel small molecules through a circadian screen of a structurally diverse library of ∼120,000 uncharacterized compounds. We found a purine derivative, longdaysin, that dramatically lengthens the circadian period. Identification and characterization of longdaysin-target proteins revealed the roles for protein kinases CKIα and ERK2 in period regulation, as well as confirmed the importance of CKIδ. Simultaneous inhibition of these three kinases drastically lengthened the circadian period, illustrating a new facet of the clock mechanism whose robustness is conferred in part by a multiple kinase network.

## Results

### Identification of a Novel Compound Lengthening the Circadian Period

**Figure 1:**
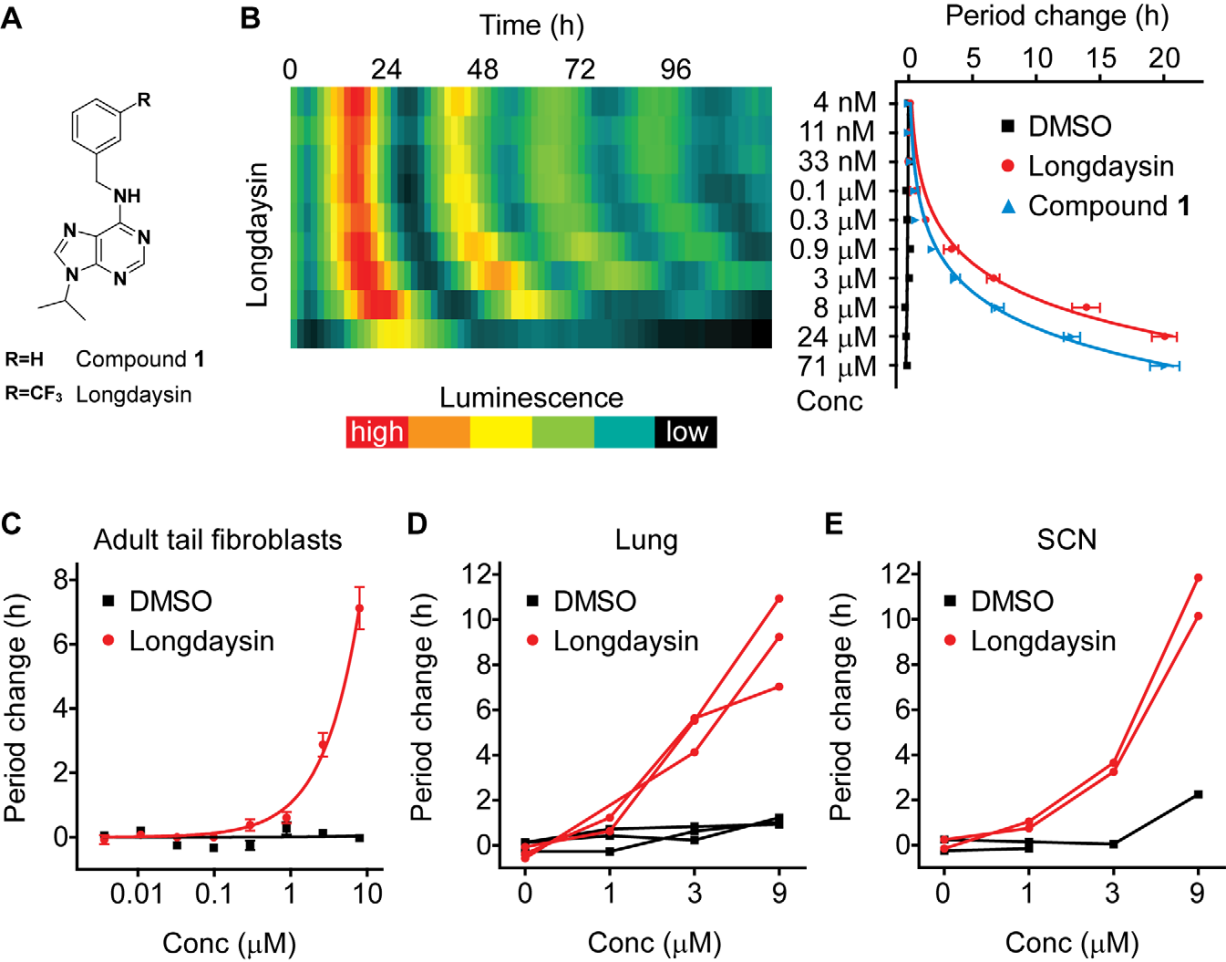
Effect of a novel compound on the circadian period in cultured cells and tissues. (A) The chemical structure of compound **1** and longdaysin. (B) Effects of compound **1** and longdaysin on the luminescence rhythms in *Bmal1-dLuc* U2OS cells. Luminescence rhythms were monitored in the presence of various concentrations of compound (10 points of 3-fold dilution series in DMSO; final 0.7% DMSO). The representative luminescence profiles for longdaysin treatment are indicated as raster plot (left panel), in which each horizontal raster line represents a single well, with elapsed time plotted to the right. Luminescence intensity is indicated by color scale. Period parameter was obtained by curve fitting, and period change relative to the mean of DMSO control was plotted against compound concentration (right panel; the mean with SEM, *n* = 4). Longdaysin showed cytotoxicity at 71 µM. (C–E) Effect of longdaysin on the circadian period in adult tail fibroblasts (C), lung explants (D), and SCN explants (E) from *mPer2^Luc^* knockin mice. Fibroblasts were cultured in the presence of various concentrations of longdaysin (the mean with SEM, *n* = 4). Lung and SCN explants were cultured in the presence of increasing concentration of longdaysin (0 to 9 µM; 1 wk for each concentration), and period change relative to the mean of DMSO control at first week was plotted for individual culture (*n* = 3 for lung and *n* = 2 for SCN).

By applying a high-throughput circadian assay system using human U2OS cells with *Bmal1-dLuc* reporter [29], we analyzed approximately 120,000 uncharacterized compounds corresponding to diverse chemical scaffolds [43]–[44] at a final concentration of 7 µM. We identified a number of compounds with different scaffolds that lengthened the circadian period of cellular luminescence rhythms. Among them, we selected one purine derivative compound **1** (Figure 1A) for follow-up studies, because it strongly lengthened the period in a dose-dependent manner and showed less effect on the amplitude of *Bmal1-dLuc* rhythms (Figure S1). A preliminary structure-activity relationship study helped to identify a derivative of compound **1** that is 3 times more potent and able to generate >10 h period change at a concentration of 10 µM (Figure 1B and Table 1). We termed this derivative “longdaysin” (Figure 1A), based on its prominent period lengthening effect. We further investigated the effect of longdaysin (Figure 1C–E) by using primary cells and tissues isolated from *mPer2^Luc^* knockin mice harboring a *mPer2^Luc^* reporter [45]–[46] as an additional clock-controlled reporter different from *Bmal1-dLuc* used in the screen. Longdaysin consistently caused dose-dependent period lengthening in adult tail fibroblasts (Figure 1C) and lung explants (Figure 1D), which represent peripheral clocks, and in SCN explants (Figure 1E), which represent the central clock. The effect of longdaysin was reversible, as the period length returned to normal after washout of the compound (Figure S2). Taken together, these results demonstrate that longdaysin potently lengthens the circadian period in multiple mammalian cells including SCN neurons.

**Table 1:** Effective concentrations of longdaysin and compound 1.

	U2OS Cell-Based Circadian Assay (Concentrations for Period Change, µM)^a^			In Vitro Kinase Assay (IC_50_, µM)^b^				Cell-Based PER1 Degradation Assay (EC_50_, µM)^c^	
Compound	5 h	10 h	15 h	CKIδ	CKIα	ERK2	CDK7	CKIδ	CKIα
Longdaysin	1.5	5.7	13	8.8	5.6	52	29	9.7	9.2
Compound 1	4.4	17	38	21	23	160	29	n.d.	n.d.

**a–c:** Values were determined from

a Figure 1B,

b Figure 2E, and

c Figure 4E.

n.d., not determined.

### Binding of Longdaysin With Protein Kinases

**Figure 2:**
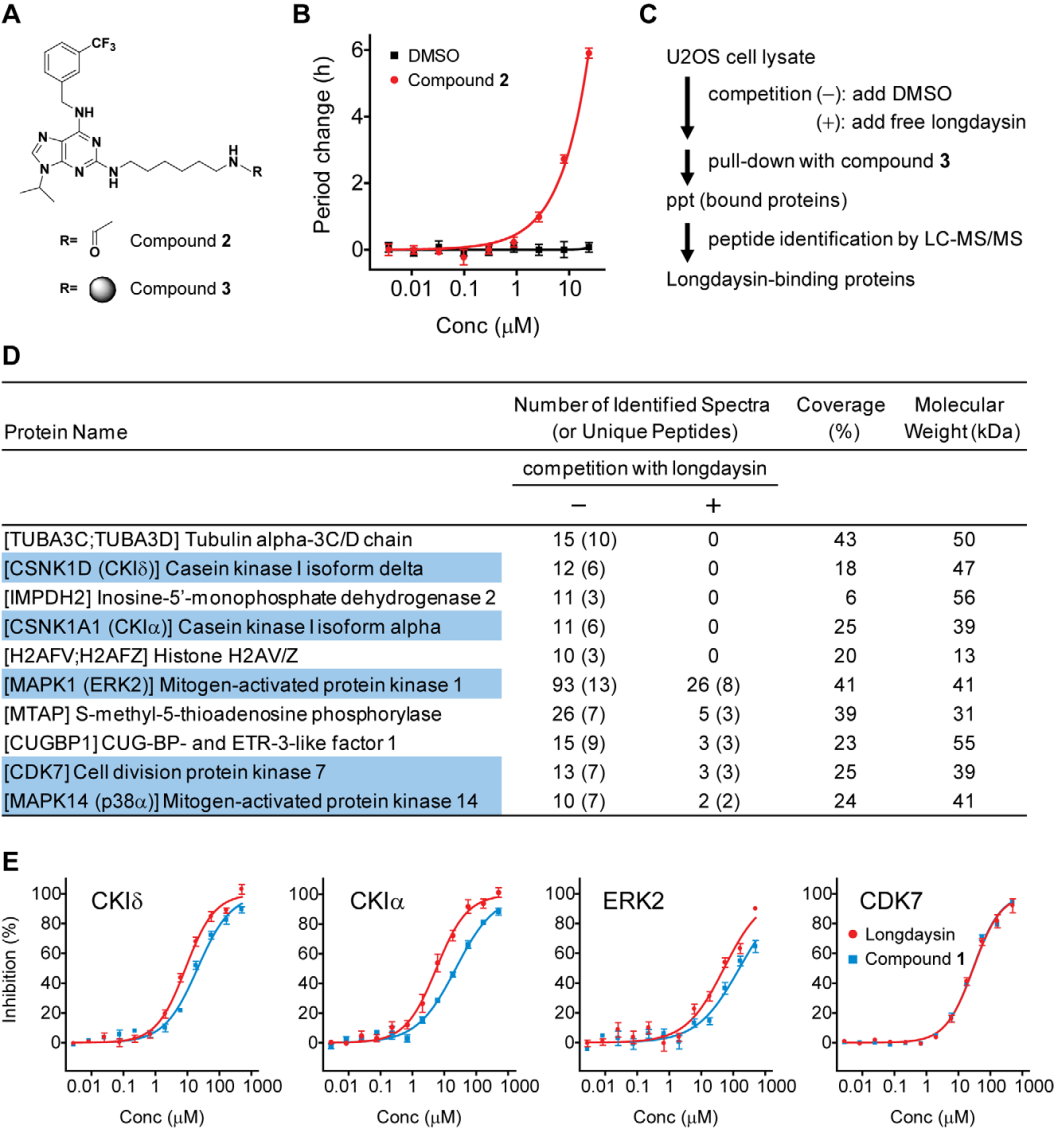
Identification of potential longdaysin-binding proteins. (A) The chemical structure of compounds **2** and **3**. A circle represents an agarose bead. (B) Effect of compound **2** on the circadian period. Luminescence rhythms of *Bmal1-dLuc* U2OS cells were monitored in the presence of various concentrations of compound **2**. Data are the mean with SEM (*n* = 4). (C) The scheme of longdaysin-binding protein identification. Cell lysate was prepared from confluent and unsynchronized U2OS cells. (D) A list of potential longdaysin-binding proteins. Listed proteins were identified by ≥10 tandem MS spectra and showed ≥3-fold signal reduction upon competition with free longdaysin. Protein kinases are highlighted in blue. Sequence coverage refers to the experiment performed in the absence of free longdaysin [competition (−)]. (E) Effects of longdaysin and compound **1** on protein kinase activity. Activities of CKIδ, CKIα, ERK2, and CDK7 in vitro were analyzed in the presence of various concentrations of compound. Data are the mean with SEM (*n* = 6 for longdaysin, *n* = 4 for compound **1**).

In order to identify potential biological targets of longdaysin by affinity-based proteomic approaches [38], we synthesized longdaysin analogs with an aminohexyl linker, based on the preliminary structure-activity relationship analysis. Among them, compound **2** with a linker at the C2 position (Figure 2A) retained the period lengthening effect in the cell-based circadian assay (Figure 2B). We then prepared agarose-conjugated compound **3** (Figure 2A) and incubated it with U2OS cell lysate in the presence or absence of 100 µM longdaysin as a soluble competitor (Figure 2C). Proteins that bound to the affinity resin, and could be competed off by free longdaysin, were separated by SDS-PAGE and analyzed by liquid chromatography-tandem mass spectrometry (LC-MS/MS). This analysis yielded 10 proteins (Figure 2D) including the protein kinases (highlighted in blue) CKIδ (CSNK1D), CKIα (CSNK1A1), ERK2 (MAPK1), CDK7, and p38α (MAPK14). Independent affinity chromatography followed by Western blotting with specific antibodies confirmed both the binding of the protein kinases to the affinity resin as well as decreased binding in the presence of free longdaysin (Figure S3). Furthermore, in vitro kinase assays revealed that longdaysin inhibited CKIδ, CKIα, ERK2, and CDK7 activities (IC_50_ = 8.8, 5.6, 52, and 29 µM, respectively; Figure 2E and Table 1), while it had much less effect on p38α (unpublished data). In contrast, compound **1** inhibited CKIδ, CKIα, and ERK2 with ∼3 times less potency than longdaysin and inhibited CDK7 similarly to longdaysin (Figure 2E and Table 1). The difference in potency between longdaysin and compound **1** against CKIδ, CKIα, and ERK2 was consistent with their cellular period effects (Table 1), suggesting an involvement of these three kinases in the period regulation.

### Lengthening of the Circadian Period by Knockdown of CKIδ, CKIα, and ERK2

**Figure 3:**
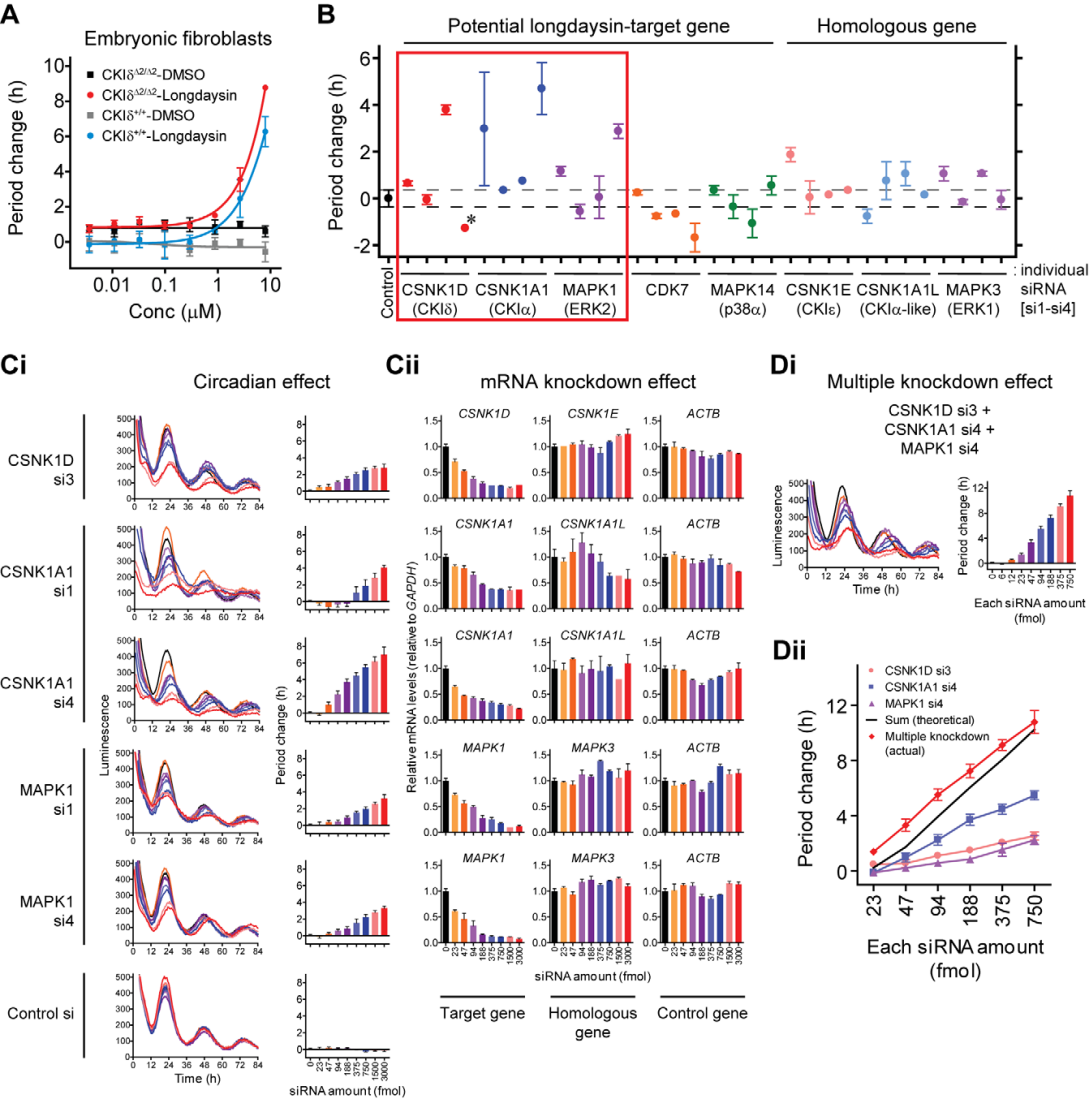
Effect of kinase gene knockdown on the circadian period. (A) Effect of longdaysin on the circadian period in embryonic fibroblasts from CKIδ deficient *mPer2^Luc^* knockin mice. Luminescence rhythms of CKIδ deficient (CKIδ^Δ2/Δ2^) or wild type (CKIδ^+/+^) cells were monitored in the presence of various concentrations of longdaysin. Period change relative to the mean of DMSO control of wild type cells was plotted (the mean with SEM, *n* = 4). (B) Effects of kinase gene siRNAs on the circadian period in *Bmal1-dLuc* U2OS cells. Luminescence rhythms were monitored after transient transfection with siRNA. Period parameter was obtained by curve fitting, and period change relative to the mean of control was plotted. Data are the mean with variation (*n* = 2). The period estimation for CSNK1D si4 was not accurate because of poor curve fitting (indicated by asterisk). (C) Dose-dependent effects of kinase gene siRNAs on the circadian period (Ci) and the gene expression (Cii). Luminescence rhythms of *Bmal1-dLuc* U2OS cells were monitored after transient transfection with various amounts of siRNA (Ci). Representative profiles are indicated in the left panels, and period changes were plotted against siRNA amount in the right panels (the mean with SEM, *n* = 5–6). Gene expression of unsynchronized cells at time 0 h was analyzed by RT-qPCR (Cii). Expression levels of target gene, homologous gene, and control gene (*ACTB*) are indicated in the left, middle, and right panels, respectively, by setting control value as 1. Data are the mean with variation (*n* = 2). (Di) Effect of multiple kinase gene knockdown on the circadian period. Equal amounts of CSNK1D si3, CSNK1A1 si4, MAPK1 si4, and control siRNA were mixed and used for transient transfection. Data are the mean with SEM (*n* = 8). (Dii) Comparison of single and multiple kinase gene knockdown effects. Effects of single (pink, blue, and purple lines; data from Ci) and multiple (red line; data from Di) gene knockdown were plotted against each siRNA amount. Black line is the theoretical sum of single gene knockdown effects.

To identify the protein(s) mediating longdaysin effect on period length, we first tested the contribution of CKIδ, a well-characterized kinase in period regulation [12],[18]–[19],[47], by using embryonic fibroblasts prepared from CKIδ deficient mice harboring the *mPer2^Luc^* knockin reporter [18]. In a 384-well plate format, the period of CKIδ deficient (CKIδ^Δ2/Δ2^) cells was 1.1 h longer than that of wild type (CKIδ^+/+^) cells (CKIδ^Δ2/Δ2^, 25.9±0.5 h; CKIδ^+/+^, 24.8±0.9 h; *n* = 48), consistent with a previous report [18]. We found that longdaysin lengthened the period in a dose-dependent manner in CKIδ deficient cells as well as in wild type cells (Figure 3A). This result indicates the presence of additional longdaysin-target(s) that regulate period length besides CKIδ.

To investigate the effects of RNAi-mediated inhibition of potential longdaysin-targeted kinases on the circadian period, we conducted knockdown experiments by applying four independent siRNAs against each gene. At least two siRNAs for *CSNK1D* (encoding CKIδ), *CSNK1A1* (CKIα), and *MAPK1* (ERK2) caused period lengthening (Figure 3B, red box), while those for *CDK7* and *MAPK14* (p38α) did not, thus proposing CKIδ, CKIα, and ERK2 as the potential clock-acting targets of longdaysin. We also tested siRNAs against the close homologs of these three kinases (*CSNK1E* for *CSNK1D*, *CSNK1A1L* for *CSNK1A1*, and *MAPK3* for *MAPK1*) and found that the homologs had little or no effect on the period (Figure 3B). The minor period effects for *MAPK14*, *CSNK1E*, and *CSNK1A1L* are in line with previous reports [23],[42]. We further looked at the primary screening data from our genome-wide RNAi study [33] in which we used four siRNAs different from this study by combining two siRNAs as a pool. Among the 10 longdaysin-interacting proteins identified by the affinity chromatography (Figure 2D), only *CSNK1D*, *CSNK1A1*, and *MAPK1* showed period lengthening of the reporter with both siRNA pairs (Figure S4, red box), supporting important roles for CKIδ, CKIα, and ERK2 in period regulation as longdaysin targets. We then characterized the period lengthening effects of siRNAs against *CSNK1D*, *CSNK1A1*, and *MAPK1* by using an 8-point dilution series of the effective siRNAs. All siRNAs tested gave dose-dependent changes of the period (Figure 3Ci) and reduction of the target gene mRNA levels without affecting the levels of closely homologous genes (Figure 3Cii). The only exception was CSNK1A1 si1, which has sequence similarity against *CSNK1A1L* mRNA (96% identical on a 23 bp stretch) and reduced its level at higher dose (Figure 3Cii). The correlation between period effect and mRNA knockdown effect matched well for two siRNAs against *CSNK1A1* or *MAPK1* (Figure S5). The proportional changes of circadian function by dose-dependent knockdown of *CSNK1D*, *CSNK1A1*, and *MAPK1* (Figure 3C) are common characteristics among the core clock components and clock modifiers [23],[33]. Taken together, these results illustrate the involvement of CKIα and ERK2 in the period regulation, as well as confirming the importance of CKIδ.

We further tested knockdown of all three kinases *CSNK1D*, *CSNK1A1*, and *MAPK1* in combination, in order to determine if their concomitant reduction could explain the strong effect of longdaysin as an inhibitor of all three kinases. Combinatorial knockdown of the three genes caused strong and dose-dependent lengthening of the period to >10 h (Figure 3Di). The multiple gene knockdown effect (Figure 3Dii, red line) matched well with the theoretical sum of the effect of single gene knockdown (black line). These results suggest that the knockdown of these three kinases works in an additive manner to cause prominent period lengthening similar to that generated by longdaysin.

### CKIα-Dependent Phosphorylation and Degradation of PER1

**Figure 4:**
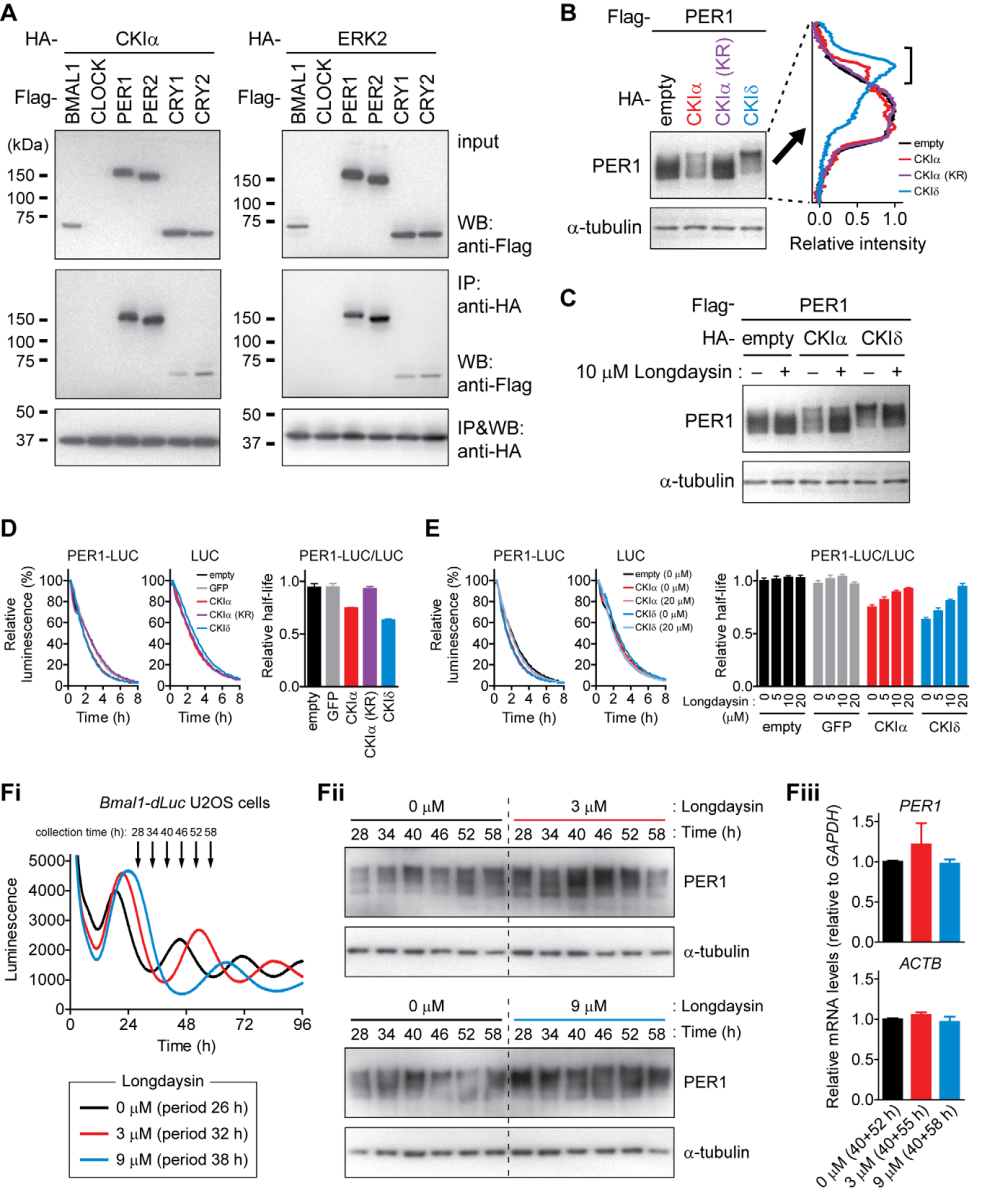
Effect of CKIα on the core clock proteins. (A) Interaction of CKIα and ERK2 with the core clock proteins. HA-tagged CKIα or ERK2 was co-expressed with Flag-tagged clock proteins in HEK293T cells and subjected to immunoprecipitation assay with anti-HA antibody. Because of the low level expression CLOCK, its interaction could not be tested. The interaction of ERK2 with CRY1 and CRY2 was detected as reported previously [67]. (B) CKIα-dependent phosphorylation of PER1. PER1 was co-expressed with CKIα, kinase-dead mutant of CKIα [CKIα (KR)], or CKIδ in HEK293T cells and analyzed by Western blot 2 d after transfection (left panel). Intensity profile of each PER1 band is shown in right panel by setting the peak value as 1. Bracket indicates lower electrophoretic mobility forms of PER1 whose intensity increased in the presence of CKIα or CKIδ. (C) Effect of longdaysin on CKIα- and CKIδ-dependent phosphorylation of PER1. PER1 was co-expressed with CKIα or CKIδ in HEK293T cells. The cells were treated with 10 µM longdaysin or 0.1% DMSO for 24 h, and a part of the cell extract was analyzed by Western blot. (D) Effect of CKIα on PER1 stability. Luciferase-fused PER1 (PER1-LUC) or luciferase (LUC) was co-expressed with GFP, CKIα, CKIα (KR), or CKIδ in HEK293T cells. The cells were treated with cycloheximide from time 0, and luminescence was recorded. Representative profiles for PER1-LUC and LUC are shown in the left and middle panels, respectively, by setting peak luminescence as 100%. Half-life of PER1-LUC was divided by that of LUC to cancel out the effect of LUC degradation on PER1-LUC stability (right panel). Data are the mean with SEM (*n* = 4). (E) Effect of longdaysin on CKIα- and CKIδ-dependent regulation of PER1 stability. PER1-LUC or LUC was co-expressed with GFP, CKIα, or CKIδ in HEK293T cells. The cells were treated with 0 to 20 µM longdaysin for 24 h and then treated with cycloheximide for luminescence recording. Representative profiles for PER1-LUC and LUC are shown in the left and middle panels, respectively. The relative half-life of PER1-LUC against LUC is indicated (right panel). Data are the mean with SEM (*n* = 6). (F) Effect of longdaysin on endogenous PER1 level in *Bmal1-dLuc* U2OS cells. Luminescence rhythms were monitored in the presence of 0, 3, or 9 µM longdaysin (Fi). In parallel, the cells were collected at indicated time points and analyzed by Western blotting (Fii). Also, the cells were collected at two time points separated by about half of the period length (40 and 52 h for 0 µM longdaysin, 40 and 55 h for 3 µM, 40 and 58 h for 9 µM) and analyzed by RT-qPCR as a mixture of the two time points (Fiii, the mean with SEM, *n* = 4).

In contrast to the well-characterized roles of CKIδ/ε-mediated phosphorylation of PER proteins [9]–[21], the functions of CKIα and ERK2 in period regulation have yet to be characterized. To examine the interaction of CKIα and ERK2 with the core clock proteins, we co-expressed HA-tagged kinases with Flag-tagged clock proteins in HEK293T cells. Immunoprecipitation assay revealed interactions of both CKIα and ERK2 with PER1 and PER2, and to a lesser extent, CRY1 and CRY2 (Figures 4A, S6A, and S6B). Co-expression of PER1 with CKIα generated lower electrophoretic mobility forms of PER1 (Figure 4B), which disappeared upon phosphatase treatment (Figure S7A), suggesting CKIα-dependent phosphorylation of PER1. This modification relied on the kinase activity of CKIα, because the kinase-dead K46R mutant of CKIα [CKIα (KR)] [48] did not cause the mobility-shift of PER1 (Figure 4B). In contrast, ERK2 showed no detectable effect on the PER1 mobility (unpublished data). Treatment of the cells with longdaysin reduced the CKIα- and CKIδ-dependent mobility-shift of PER1 (Figures 4C, S7B, and S7C), consistent with a potential mode of longdaysin action through CKIα and CKIδ.

As phosphorylation of PER1 modulates its stability [16]–[17],[21], we further tested the effect of CKIα on the stability of PER1 by expressing luciferase-fused PER1 protein (PER1-LUC) in HEK293T cells. Luminescence changes were monitored following inhibition of *de novo* protein synthesis by cycloheximide treatment. Co-expression of CKIα but not CKIα (KR) accelerated PER1-LUC degradation relative to that of LUC (Figure 4D). The CKIα- and CKIδ-dependent degradation of PER1 was inhibited by longdaysin treatment in a dose-dependent manner (Figure 4E). Similar results were obtained by using PER1 without the LUC fusion (Figure S8A). In contrast, we found that CKIα had no effect on the stability of a PER2-LUC fusion protein while CKIδ accelerated its degradation (Figure S8B). These results demonstrated the selectivity of CKIα against PER1 degradation over PER2. Longdaysin inhibited the CKIδ-mediated PER2-LUC degradation in a dose-dependent manner (Figure S8C), suggesting its role in regulating both PER1 and PER2 stabilities through CKIδ and/or CKIα.

We then investigated the effect of longdaysin on the protein level of endogenous PER1 in *Bmal1-dLuc* U2OS cells during circadian cycles. The cells were synchronized with medium change and collected every 6 h from 28 h to 58 h after the medium change (Figure 4Fi). Consistent with the longdaysin-dependent shift of the second trough of *Bmal1-dLuc* luminescence rhythm (34, 38, and 46 h for 0, 3, and 9 µM longdaysin, respectively; Figure 4Fi), the second peak of PER1 protein rhythm shifted in parallel (40, 40–46, and 52 h for 0, 3, and 9 µM longdaysin, respectively; Figure 4Fii). Furthermore, 3 or 9 µM longdaysin treatment strongly up-regulated overall protein amount of PER1 compared with 0 µM control (Figure 4Fii) without affecting its mRNA level (Figure 4Fiii), demonstrating post-transcriptional increase of endogenous PER1 by longdaysin. The progressive phosphorylation of PER1 was still observed in the presence of longdaysin (Figure 4Fii), possibly because of the phosphorylation by kinase(s) that was not affected by longdaysin. Collectively, these results provide a possible mechanism of longdaysin action for period regulation through the CKIα- and CKIδ-mediated control of PER1 stability.

### Period Lengthening of Gene Expression Rhythms by Longdaysin in Zebrafish In Vivo

**Figure 5:**
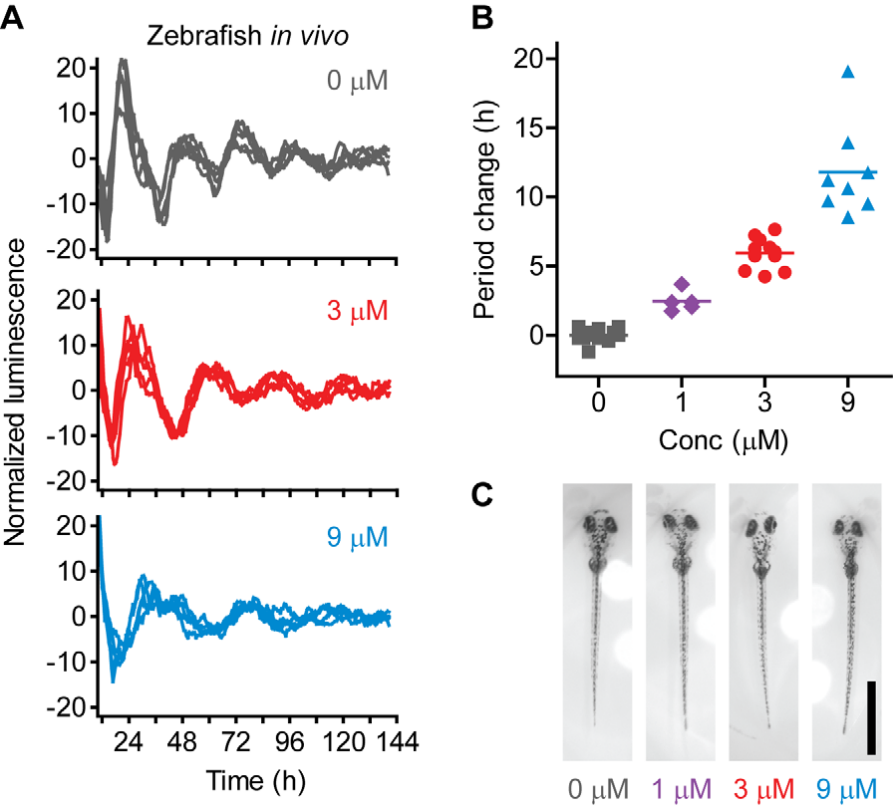
Effect of longdaysin on the circadian period in zebrafish in vivo. Zebrafish hemizygous for *per3-luc* were entrained in 12 h light/12 h dark cycles, and then luminescence rhythms were monitored in constant darkness in the presence of various concentrations of longdaysin. Representative luminescence rhythm of individual fish (*n* = 5 for each condition) was plotted after smoothing with 2.4 h moving average and baseline subtraction with fourth order polynomial curve (A). Period parameter was obtained by curve fitting and plotted against compound concentration (B) (*n* = 13, 5, 11, and 8 for 0, 1, 3, and 9 µM longdaysin, respectively). Representative pictures of zebrafish after 1 wk of treatment with longdaysin are shown (C). Scale bar, 1 mm.

Lastly, we investigated if longdaysin had any in vivo efficacy by using zebrafish, which provide a useful model system for studies on circadian rhythms at the level of the whole organism [49], and have conserved CKI and ERK family genes [50]–[51]. By using transgenic zebrafish harboring a *per3-luc* reporter [52], we first established an in vivo circadian assay to investigate the effects of compounds. Larval *per3-luc* fish were entrained in 12 h light/12 h dark cycles from day 3 to 6 postfertilization and then placed in an individual well of a 96-well plate to monitor luminescence rhythms under constant darkness. By using this assay, we found that longdaysin treatment caused >10 h period lengthening in a dose-dependent manner in *per3-luc* reporter fish (Figure 5A and 5B), without affecting body size (Figures 5C and S9). The in vivo period changes were similar to those observed in mammalian tissues and cells (Figure 1), showing the prominent characteristics of longdaysin as a period lengthening compound.

## Discussion

The present study highlighted the effectiveness of the chemical biology approach in dissecting circadian clock mechanisms. Our large-scale small molecule screening identified a novel compound longdaysin that exhibited a drastic effect on the circadian period of not only a variety of mammalian cells but also zebrafish in vivo. As a first attempt to determine the molecular mechanism underlying such a large period effect, we conducted affinity-based proteomics and siRNA-mediated knockdown analyses. Our results revealed CKIδ, CKIα, and ERK2 as targets of longdaysin for period regulation. Effective concentrations of longdaysin against CKIδ and CKIα in a cell-based PER1 degradation assay were similar to those in in vitro kinase assays (Table 1), suggesting efficient cell permeability of the compound. Treatment with 10 µM longdaysin consistently inhibited CKIδ and CKIα activities in vitro and their effects on PER1 phosphorylation and degradation, resulting in a 13 h period lengthening in U2OS cells. The increasing period effect at the range of 3 to 24 µM, in which longdaysin considerably inhibited ERK2 in vitro, supported the role of ERK2 at higher longdaysin concentration.

In mammals, the CKI family of Ser/Thr kinases contains seven members (α, β, γ1, γ2, γ3, δ, and ε). While the roles of CKIδ/ε in the circadian clock mechanism have been extensively studied, the inhibition of CKIδ/ε alone is insufficient to explain the drastic effect of longdaysin. Knockout of these genes has only a modest effect on period length and the effect of longdaysin was also observed in CKIδ deficient cells. We found that CKIα, in addition to CKIδ and CKIε, binds to PER1 and regulates its stability. This observation is reminiscent of the CKIα-, CKIδ-, and CKIε-mediated regulation of β-catenin and Ci, key players in the Wnt and Hedgehog signaling pathways [53]–[54]. Similar to NFAT transcription factors that are the targets of CKIα and/or CKIε [55]–[56], the CKI docking site of PER1/2 contains a FXXXF motif necessary for CKIε binding [56]. Although PER1/2 bind with CKIα/δ/ε, the affinity of CKIα is much lower than CKIδ/ε (Figure S6C). A recent study demonstrated that two amino acid residues in the CKI kinase domain cause weaker affinity of CKIα for PER1 compared with CKIε [22]. The low affinity will be advantageous to release PER proteins from CKIα for subsequent regulations, such as phosphorylation by other kinases and degradation. Disruption of the circadian rhythm in CKIδ deficient fibroblasts by overexpression of dominant negative form of CKIε [57] may be mediated also by perturbation of CKIα-dependent regulation, because of the tight binding of dominant negative CKIε with PER proteins. On the other hand, ERK1 and ERK2 MAP kinases have been well characterized in the resetting mechanism of the clock [58]. Our results demonstrated a role for ERK2 in the regulation of circadian period as well. Attenuation of the circadian rhythms in SCN explants by treatment with the MEK (ERK kinase) inhibitor U0126 [59] could potentially be explained by strong inhibition of both ERK1 and ERK2. Similar to CKIδ and CKIα, ERK2 bound to PER1/2, suggesting PER protein as a key node in phosphorylation-dependent period regulation by multiple kinases. The effect of ERK2 on PER phosphorylation and function will be addressed in future studies.

CKIδ, CKIα, and ERK2 are involved in diverse cellular processes such as cell proliferation and apoptosis [53],[60]. Consistently, CKIδ and ERK2 are required for normal development as revealed by gene knockout studies [18],[61]–[62], while CKIα deficient mice are not reported yet. In addition to the regulation of PER by these kinases, it is possible that the regulation of other clock proteins and/or changes in cellular physiology may also affect the circadian period. Therefore, it is important to identify specific residues of PER responsible for the CKIδ-, CKIα-, and ERK2-mediated period regulation. In contrast to the CKIδ-dependent progressive phosphorylation of PER1, CKIα caused a smaller mobility-shift (Figure 4B,C), suggesting a key role for site-specific phosphorylation rather than a global change of phosphorylation level. In *Neurospora*, a quantitative mass spectrometry approach identified >75 in vivo phosphorylated residues of the clock protein FRQ [63]. Interestingly, phosphorylation of two distinct regions leads to opposing effects on FRQ stability and circadian period [63]. In mammals, phosphorylation site mapping via mass spectrometry identified 21 phosphorylated Ser/Thr residues in PER2 overexpressed in HEK293 cells [14]. Identification of PER1 phosphorylation sites and characterization of the role of each residue will lead to the understanding of CKIδ-, CKIα-, and ERK2-mediated regulation of PER1 function and the circadian period. Furthermore, the phenotypic differences between PER1 and PER2 observed in CKIα-dependent regulation of stability (Figure S8B) and CKIε-mediated control of nuclear translocation [64] could be explained by comparing phosphorylation sites and their functions.

We found that combinatorial knockdown of CKIδ, CKIα, and ERK2 worked additively for prominent period lengthening (Figure 3D), similar to that caused by longdaysin. In contrast, knockout of CKIδ (Figure 3A), knockdown of single kinase (Figure 3B,C), and CKI inhibitors D4476 and IC261(Figure S10) all showed smaller period effects. These observations indicate that the network of multiple kinases confers robustness to the clock mechanism. A single small molecule such as longdaysin inhibiting the multiple pathways simultaneously can significantly perturb the clock system and elicit unexpectedly long period. Previous screening of the LOPAC chemical library identified several kinase inhibitors that cause large period lengthening [29],[42]. These compounds have the potential to inhibit CKIδ/ε [39]–[40],[42], although their primary target is CDK, p38 MAPK, JNK, CK2, or VEGFR signaling pathway. Because of the high conservation of the kinase domain between CKIδ and CKIα, these compounds are also likely to inhibit CKIα. Considering our current finding, the inhibition of the primary target (CDK, p38 MAPK, JNK, CK2, or VEGFR signaling pathway) in combination with CKIδ/ε/α may be essential for the large period effect of these compounds. Supporting this idea, CK2 acts cooperatively with CKIε to regulate PER2 stability [32]. Having multiple targets might be a common characteristic of therapeutically effective compounds, such as sunitinib and sorafenib for cancer treatment [65]. Our zebrafish experiments clearly showed an in vivo effect of longdaysin in a vertebrate, and further optimization of longdaysin in mammalian systems may provide a chemical starting point for the identification of small molecule therapeutics specifically designed for ameliorating circadian disorders.

## Materials and Methods

### Ethics Statement

All animal studies were approved by the University of California, San Diego, Institutional Animal Care and Use Committee and performed in accordance with the guidelines.

### Compounds

Synthesis of compound **1**, longdaysin, compound **2**, and compound **3** is described in Text S1. The dilution series of the compounds was made on 384-well plates by using a robotic liquid handling system (MiniTrak, Perkin-Elmer).

### Cell-Based Circadian Assay for Compound Experiment

The compound screen was done with the high-throughput circadian assay system as described previously [29]. In brief, *Bmal1-dLuc* U2OS cells were suspended in the culture medium (DMEM supplemented with 10% fetal bovine serum, 0.29 mg/ml L-glutamine, 100 units/ml penicillin, and 100 µg/ml streptomycin) and plated onto 384-well white solid-bottom plates at 20 µl (2,000 cells) per well. After 2 d, 50 µl of the explant medium (DMEM supplemented with 2% B27, 10 mM HEPES, 0.38 mg/ml sodium bicarbonate, 0.29 mg/ml L-glutamine, 100 units/ml penicillin, 100 µg/ml streptomycin, 0.1 mg/ml gentamicin, and 1 mM luciferin, pH 7.2) was dispensed to each well, followed by the application of 500 nl of compounds (dissolved in DMSO; final 0.7% DMSO). The plate was covered with an optically clear film and set to luminescence monitoring system equipped with a CCD imager (ViewLux, Perkin Elmer). The luminescence was recorded every 2 h for 3–4 days. In follow-up studies, the luminescence was recorded every 100 min by using a microplate reader (Infinite M200, Tecan). The period parameter was obtained from the luminescence rhythm by curve fitting program CellulaRhythm [29] or MultiCycle (Actimetrics), both of which gave similar results.

Luminescence rhythms of adult tail fibroblasts [46] and embryonic fibroblasts [18] from *mPer2^Luc^* knockin mice were analyzed similarly to U2OS cells, except that 1,800 cells were plated per well. Because of the low luminescence intensity of the fibroblasts, the higher sensitivity ViewLux imager was used for rhythm recording.

### Explant Culture of Mouse Tissues

Explants of lung and SCN were dissected from *mPer2^Luc^* knockin mice [45] and cultured in explant medium as described previously [46]. The medium was changed every week with increasing concentration of longdaysin each time (from 0 to 9 µM, final 0.7% DMSO). The luminescence was recorded every 10 min with LumiCycle luminometer (Actimetrics), and the period parameter was obtained by using LumiCycle Analysis software (Actimetrics).

### Affinity Chromatography

U2OS cells kept in confluence (2×10^8^ cells) were collected with ice-cold PBS and homogenized by using Dounce homogenizer in 5 ml of lysis buffer (25 mM MOPS, 15 mM EGTA, 15 mM MgCl_2_, 1 mM DTT, 60 mM β-glycerophosphate, 15 mM p-nitrophenyl phosphate, 1 mM Na_3_VO_4_, 1 mM NaF, 1 mM phenyl phosphate, 10 µg/ml leupeptin, 10 µg/ml aprotinin, 10 µg/ml soybean trypsin inhibitor, 100 µM benzamidine, pH7.2). The homogenate was sonicated and centrifuged (16,000×g) at 4°C for 20 min. The resulting supernatant was split into two, and each portion was incubated with or without 100 µM longdaysin (final 0.1% DMSO) at 4°C for 10 min (Figure 2C). Then, 120 µl of compound **3** [50% slurry in bead buffer (50 mM Tris, 250 mM NaCl, 5 mM EDTA, 5 mM EGTA, 0.1% NP-40, 5 mM NaF, 10 µg/ml leupeptin, 10 µg/ml aprotinin, 10 µg/ml soybean trypsin inhibitor, 100 µM benzamidine, pH 7.4)] was added to the mixture and incubated at 4°C for 1 h with rotation. The agarose beads were washed 6 times with 2 ml of the bead buffer. The bound proteins were eluted with SDS sample buffer and separated by SDS-PAGE (4%–12% gradient gel, Invitrogen). The gel was CBB stained, and the gel lane for each condition was cut horizontally into 24 pieces.

### Protein Mass Spectrometry

All gel bands were subjected to LC-MS/MS analysis as described previously [66]. Tandem MS data were analyzed using Sequest (ThermoFinnigan, San Jose, CA; Version 3.0). Sequest was set up to search a *Homo sapiens* subset of the EBI-IPI database (Version 3.32) to which a reversed copy of the protein database was appended, assuming the digestion enzyme trypsin. Sequest was searched with a fragment ion mass tolerance of 0 Da and a parent ion tolerance of 3.0 Da. Iodoacetamide derivative of cysteine was specified in Sequest as a fixed modification. Oxidation of methionine was specified in Sequest as a variable modification.

Scaffold (version Scaffold_2_05_00, Proteome Software Inc., Portland, OR) was used to validate MS/MS based peptide and protein identifications. Peptide identifications were accepted if they could be established at greater than 95.0% probability as specified by the Peptide Prophet algorithm. Protein identifications were accepted if they could be established at greater than 99.0% probability and contained at least three unique peptides. Protein probabilities were assigned by the Protein Prophet algorithm. Crude differential quantitation of proteins identified in both pulldown experiments was performed by comparing the number of assigned peptides.

### In Vitro Kinase Assay

The CKIδ, CKIα, CDK7, and ERK2 kinase assays were performed on 384-well plates (10 µl volume). The reaction mixture was as follows: for CKIδ, 2 ng/µl CKIδ (Millipore, 14-520), 50 µM peptide substrate RKKKAEpSVASLTSQCSYSS corresponding to human PER2 Lys659-Ser674 [47], and CKI buffer (40 mM Tris, 10 mM MgCl_2_, 0.5 mM DTT, 0.1 mg/ml BSA, pH 7.5); for CKIα, 1 ng/µl CKIα (Invitrogen, PV3850), 50 µM CKI peptide substrate (Anaspec, 60547-1), and CKI buffer; for CDK7, 5 ng/µl CDK7 (Millipore, 14-476), 100 µM Cdk7/9 peptide substrate (Millipore, 12-526), and CKI buffer; for ERK2, 1.5 ng/µl ERK2 (Millipore, 14-550), 0.8 µg/µl MBP (Millipore, 13-104), and ERK buffer (50 mM Tris, 10 mM MgCl_2_, 0.5 mM DTT, 1 mM EGTA, pH 7.5). Five hundred nl of compound was added to the mixture (final 5% DMSO), and the reaction was started by adding ATP (final 5 µM). After incubation at 30°C for 3h, 10 µl of Kinase-Glo Luminescent Kinase Assay reagent (Promega) was added, and the luminescence was detected to determine remaining ATP amount. All of the tested compounds did not inhibit luciferase activity directly. IC_50_ value was obtained by using Prism software (GraphPad Software).

### Cell-Based Circadian Assay for RNAi Experiment

siRNAs against protein kinase genes (obtained from Human Protein Kinome Set, Integrated DNA Technologies) were tested on 384-well plates in Figure 3B, and resynthesized siRNAs (Table S1, Integrated DNA Technologies) were tested on 96-well plates in Figure 3C,D by using *Bmal1-dLuc* U2OS cells as described previously [29],[33]. In brief, for 96-well plates, the siRNA was spotted onto white solid-bottom plates, and 60 µl of Opti-MEM (Invitrogen) containing 0.4 µl of Lipofectamine 2000 (Invitrogen) was dispensed onto each well. After incubation at room temperature for 20 min, 60 µl of the cells in DMEM supplemented with 20% fetal bovine serum was dispensed (6,000 cells/well). The cells were cultured overnight, and the medium was changed to 180 µl of the culture medium. After 2 d, the medium was changed to 180 µl of the explant medium, and the plate was covered with optically clear film. The luminescence was recorded every 36 min by using the Tecan luminometer. The period parameter was obtained from the luminescence rhythm by using MultiCycle software.

### RT-qPCR

*Bmal1-dLuc* U2OS cells were transfected with siRNAs as described above and harvested just before the change to the explant medium (i.e., the cells were unsynchronized at the time of harvest). Total RNA preparation and RT-qPCR were performed as described previously [29],[33]. The primers for qPCR are listed in Table S2.

### Transient Transfection and Immunoprecipitation

HEK293T cells (1.25×10^6^ cells) were reverse transfected on 6-well plates by Lipofectamine 2000 with 1 µg each of expression vectors for C-terminally 3XFlag-tagged clock protein (in p3XFLAG-CMV-14, Sigma) and N-terminally HA-tagged kinase (in p3XFLAG-CMV-14). For ERK2, 0.05 µg of expression vector with 0.95 µg of empty vector was used because of its efficient expression. After 24 h, the cells were collected with ice-cold PBS and suspended in 100 µl of incubation buffer [50 mM Tris, 50 mM NaCl, 2 mM EDTA, 10% glycerol, 1 mM DTT, Complete Protease Inhibitor Cocktail (Roche), Phosphatase Inhibitor Cocktail 1 and 2 (Sigma), pH 8.0]. The mixture was supplemented with NP-40 (final 1%) and incubated on ice for 15 min, followed by centrifugation (16,000×g) at 4°C for 10 min. A part of the resulting supernatant (40 µl) was incubated with 0.4 µg of anti-HA antibody (Roche, 11867423001) cross-linked with Dynabeads Protein G (Invitrogen) at 4°C for 2 h with rotation. The beads were washed twice with the incubation buffer supplemented with 1% NP-40. The bound proteins were eluted with SDS sample buffer, separated by SDS-PAGE (4%–12% gradient gel), and analyzed by Western blotting with anti-Flag antibody (Sigma, F1804) or anti-HA antibody conjugated with HRP (Roche, 12013819001). For the analysis of PER1 electrophoretic mobility-shift, the cell extracts were separated by SDS-PAGE (3%–8% gradient gel, Invitrogen) and analyzed by Western blotting with anti-Flag antibody or anti-α-tubulin antibody (Santa Cruz Biotechnology, sc-32293). Protein concentration of each sample was measured by the Lowry method using DC protein assay (BioRad).

### Protein Degradation Assay

HEK293T cells (6.0×10^4^ cells) were reverse transfected on 96-well white solid-bottom plates by Lipofectamine 2000 with 40 ng each of expression vectors for C-terminally luciferase-fused PER1 (in p3XFLAG-CMV-14) and N-terminally HA-tagged kinase (in p3XFLAG-CMV-14). For luciferase (in p3XFLAG-CMV-14), 2 ng of expression vector with 38 ng of empty vector was used because of its efficient expression. After 48 h, the medium was supplemented with luciferin (final 1 mM) and HEPES-NaOH (pH 7.2; final 10 mM). After 1 h, cycloheximide (final 20 µg/ml) was added to the medium, and the plate was covered with optically clear film. The luminescence was recorded every 10 min by using the Tecan luminometer. Half-life was obtained by using Prism software (GraphPad Software).

### Time-Course Assay of Endogenous PER1 Abundance

*Bmal1-dLuc* U2OS cells were plated onto 6-well-plates (2.0×10^5^ cells/well). After 2 d, the medium was replaced with 2 ml explant medium containing 0, 3, or 9 µM longdaysin. The plate was covered with film and kept at 36°C. At indicated time points, the cells were collected with ice-cold PBS and stored at −80°C. Then the cell pellets were homogenized in SDS sample buffer and analyzed by Western blotting with anti-PER1 antibody (Cosmo Bio, KAL-KI044) or anti-α-tubulin antibody. In parallel, luminescence rhythms of the cells plated on 35 mm dishes were recorded with LumiCycle luminometer at 36°C.

### In Vivo Measurement of Luminescence Rhythms in Zebrafish

The *per3-luc* transgenic line [52] was obtained from Zebrafish International Resource Center. Hemizygote larval fish were entrained in 12 h light/12 h dark cycles from day 3 to 6 postfertilization. They were then placed in an individual well of a 96-well white solid-bottom plate with 180 µl of E3 solution (5 mM NaCl, 0.17 mM KCl, 0.33 mM CaCl_2_, and 0.33 mM MgSO_4_, pH 7.0) containing 0.5 mM luciferin, 0.013% Amquel Plus Instant Water Detoxifier (Kordon brand; Novalek, Hayward, California, United States), and various concentrations of longdaysin (final 0.1% DMSO). The plate was covered with optically clear film, and the luminescence was recorded every 36 min by using the Tecan luminometer at 25°C. The period parameter was obtained from the luminescence rhythm by using MultiCycle software.

## Supporting Information

Figure S1 **Effect of compound 1 on the luminescence rhythms in** ***Bmal1-dLuc*** **U2OS cells.** Luminescence rhythms were monitored in the presence of various concentrations of compound **1**. The representative profiles are indicated as raster plot (left panel), in which each horizontal raster line represents a single well, with elapsed time plotted to the right. Luminescence intensity is indicated by color scale. Period parameter was obtained by curve fitting, and period change relative to the mean of DMSO control was plotted against compound concentration (right panel; the mean with SEM, *n* = 4).
(0.26 MB PDF)

Figure S2 **Effect of washout of longdaysin on the circadian period in cultured cells and tissues.** *Bmal1-dLuc* U2OS cells (left panel) were cultured in the presence of various concentrations of longdaysin for 5 d (pre-wash). Then the medium was replaced, and the cells were cultured without longdaysin for another 5 d (post-wash). Period change relative to the mean of pre-wash 0 µM condition was plotted for individual culture (*n* = 5 for each condition). Adult tail fibroblasts (middle panel) and lung explants (right panel) from *mPer2^Luc^* knockin mice were treated similarly (fibroblasts, *n* = 4, 3, and 4 for 0, 3, and 9 µM, respectively; lung, *n* = 3 and 4 for 0 and 9 µM, respectively).
(0.24 MB PDF)

Figure S3 **Binding of protein kinases to longdaysin.** Affinity chromatography was performed as described in Figure 2C. Proteins bound to compound **3** were subjected to Western blotting with specific antibodies [anti-CKIδ (Santa Cruz Biotechnology, sc-55553), anti-CKIα (Cell Signaling Technology, 2655), anti-ERK1/2 (Cell Signaling Technology, 9102), and anti-CDK7 (Santa Cruz Biotechnology, sc-529)]. Anti-CKIα reacted with short (close arrowhead) and long (open arrowhead) variants of CKIα. Note that ERK1, a homolog of ERK2, showed less binding to compound **3**, indicating the selectivity of the compound.
(0.45 MB PDF)

Figure S4 **Effects of siRNAs against longdaysin-binding proteins on the circadian period.** Data are extracted from the primary screen of our genome-wide RNAi study [33]. Luminescence rhythms of *Bmal1-dLuc* U2OS cells were monitored after transient transfection with siRNAs. Four independent siRNAs were tested per gene as two siRNA pools (#1 and #2), each containing two independent siRNAs. Data are the mean with variation (*n* = 2). Asterisk indicates low amplitude rhythm, which sometimes causes wrong period estimation because of poor curve fitting. Pairs #1 and #2 for H2AFV and pair #1 for H2AFZ have highly possible off-target genes, and the result is not shown.
(0.23 MB PDF)

Figure S5 **Comparison of the effect of individual siRNA on the circadian period and the target gene expression.** Target gene knockdown effect from Figure 3Cii (*x*-axis in log scale; the mean with variation, *n* = 2) was plotted against period effect from Figure 3Ci (*y*-axis; the mean with SEM, *n* = 5–6) for each siRNA.
(0.24 MB PDF)

Figure S6 **Interaction of CKIα and ERK2 with the clock proteins.** (A) Long exposure images of Figure 4A. (B) Interaction of CKIα and ERK2 with PER1/2. (C) Interaction of CKIα, CKIδ, and CKIε with PER1/2. HA-tagged kinases were co-expressed with Flag-tagged clock proteins in HEK293T cells and subjected to immunoprecipitation assay with anti-HA antibody.
(1.35 MB PDF)

Figure S7 **Effect of longdaysin on CKIα- and CKIδ-dependent phosphorylation of PER1.** (A) HEK293T cell extract expressing PER1 and CKIα was treated with λ protein phosphatase in the absence or presence of phosphatase inhibitor and analyzed by Western blot. (B and C) PER1 was co-expressed with CKIα or CKIδ in HEK293T cells. The cells were treated with longdaysin (10 µM in B and 20 µM in C) or DMSO for 24 h and analyzed by Western blot. Intensity profile of each PER1 band is shown in (B) and bottom panels of (C) by setting the peak value as 1. Western blot image for (B) is shown in Figure 4C. Arrows indicate (sub)peaks of PER1, which appeared depending on CKIα or CKIδ and shifted by longdaysin treatment.
(0.56 MB PDF)

Figure S8 **Effect of longdaysin on CKIα- and CKIδ-dependent regulation of PER1 and PER2 stability.** (A) Flag-tagged PER1 was co-expressed with CKIα or CKIδ in HEK293T cells. The cells were treated with 0, 10, or 20 µM longdaysin for 24 h and then treated with cycloheximide from time 0. The cells were collected 0, 2, 4, or 6 h later and analyzed by Western blot. Note that co-expression of CKIα or CKIδ accelerated degradation of PER1 compared with empty vector control (upper panels, 0 µM longdaysin). The effects of CKIα and CKIδ were partially inhibited by 10 µM longdaysin (middle panels) and strongly inhibited by 20 µM longdaysin (lower panels). (B) PER1-LUC, PER2-LUC, or LUC was co-expressed with various amounts of GFP, CKIα, CKIα (KR), or CKIδ in HEK293T cells. The cells were treated with cycloheximide from time 0, and luminescence was recorded. The relative half-life of PER1-LUC (upper panel) or PER2-LUC (lower panel) against LUC is indicated. Data are the mean with SEM (*n* = 4). Note that CKIα did not affect PER2-LUC stability, while its effect on PER1-LUC was saturated at 80 ng condition. (C) PER2-LUC or LUC was co-expressed with GFP, CKIα, or CKIδ in HEK293T cells. The cells were treated with 0 to 20 µM longdaysin for 24 h and then treated with cycloheximide for luminescence recording. The relative half-life of PER2-LUC against LUC is indicated. Data are the mean with SEM (*n* = 4).
(0.96 MB PDF)

Figure S9 **Effect of longdaysin on body length of zebrafish.** Zebrafish were treated with longdaysin for luminescence recording (Figure 5). Body length of zebrafish was measured after the 1 wk treatment and plotted against compound concentration (*n* = 13, 5, 11, and 8 for 0, 1, 3, and 9 µM longdaysin, respectively).
(0.21 MB PDF)

Figure S10 **Effects of CKI inhibitors D4476 and IC261 on the circadian period.** Luminescence rhythms of *Bmal1-dLuc* U2OS cells were monitored in the presence of various concentrations of compounds. Data are the mean with SEM (*n* = 4). Longdaysin and D4476 showed cytotoxicity at 71 µM.
(0.23 MB PDF)

Table S1 **siRNA sequences.**
(0.22 MB PDF)

Table S2 **qPCR primer sequences.**
(0.24 MB PDF)

Text S1 **Supporting methods.** Synthesis of compound **1**, Longdaysin, compound **2**, and compound **3**.
(0.49 MB DOC)

## Abbreviations

FASPS: familial advanced sleep phase syndrome
LC-MS/MS: liquid chromatography-tandem mass spectrometry
LOPAC: Library of Pharmacologically Active Compounds
SCN: suprachiasmatic nucleus
