# Supplementary figures and images for: High-Throughput Chemical Screen Identifies a Novel Potent Modulator of Cellular Circadian Rhythms and Reveals CKIα as a Clock Regulatory Kinase
Source: PLoS Biol. 2010 Dec 14;8(12):e1000559. doi: 10.1371/journal.pbio.1000559 (PMC3001897; doi:10.1371/journal.pbio.1000559)

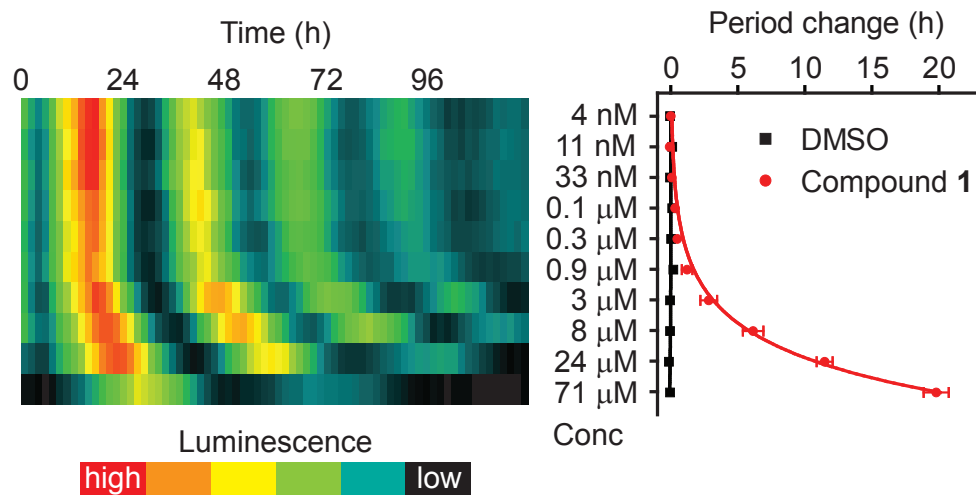

Supplement: Figure S1 — Effect of compound 1 on the luminescence rhythms in Bmal1-dLuc U2OS cells. Luminescence rhythms were monitored in the presence of various concentrations of compound 1. The representative profiles are indicated as raster plot (left panel), in which each horizontal raster line represents a single well, with elapsed time plotted to the right. Luminescence intensity is indicated by color scale. Period parameter was obtained by curve fitting, and period change relative to the mean of DMSO control was plotted against compound concentration (right panel; the mean with SEM, n = 4). (0.26 MB PDF) [file pbio.1000559.s001.pdf]

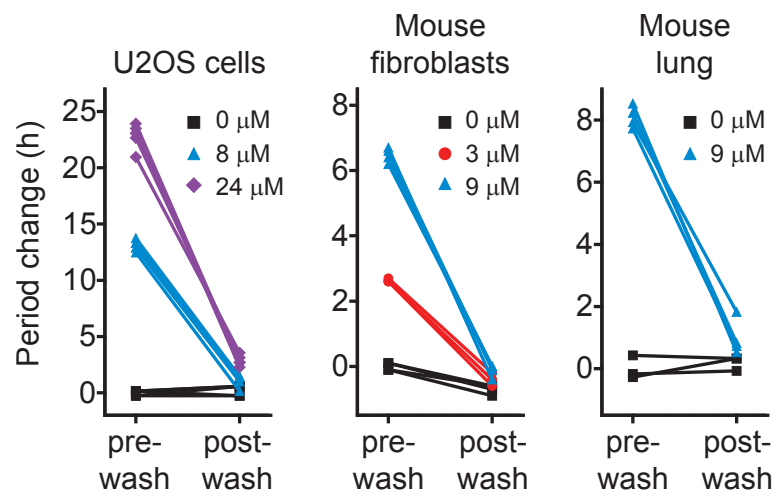

Supplement: Figure S2 — Effect of washout of longdaysin on the circadian period in cultured cells and tissues. Bmal1-dLuc U2OS cells (left panel) were cultured in the presence of various concentrations of longdaysin for 5 d (pre-wash). Then the medium was replaced, and the cells were cultured without longdaysin for another 5 d (post-wash). Period change relative to the mean of pre-wash 0 µM condition was plotted for individual culture (n = 5 for each condition). Adult tail fibroblasts (middle panel) and lung explants (right panel) from mPer2Luc knockin mice were treated similarly (fibroblasts, n = 4, 3, and 4 for 0, 3, and 9 µM, respectively; lung, n = 3 and 4 for 0 and 9 µM, respectively). (0.24 MB PDF) [file pbio.1000559.s002.pdf]

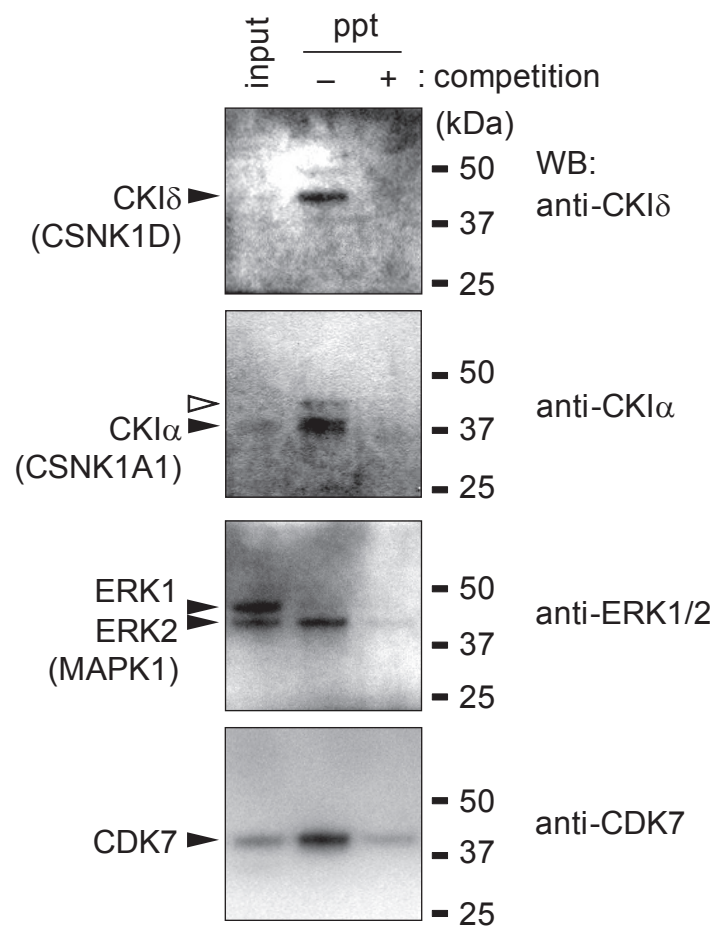

Supplement: Figure S3 — Binding of protein kinases to longdaysin. Affinity chromatography was performed as described in Figure 2C. Proteins bound to compound 3 were subjected to Western blotting with specific antibodies [anti-CKIδ (Santa Cruz Biotechnology, sc-55553), anti-CKIα (Cell Signaling Technology, 2655), anti-ERK1/2 (Cell Signaling Technology, 9102), and anti-CDK7 (Santa Cruz Biotechnology, sc-529)]. Anti-CKIα reacted with short (close arrowhead) and long (open arrowhead) variants of CKIα. Note that ERK1, a homolog of ERK2, showed less binding to compound 3, indicating the selectivity of the compound. (0.45 MB PDF) [file pbio.1000559.s003.pdf]

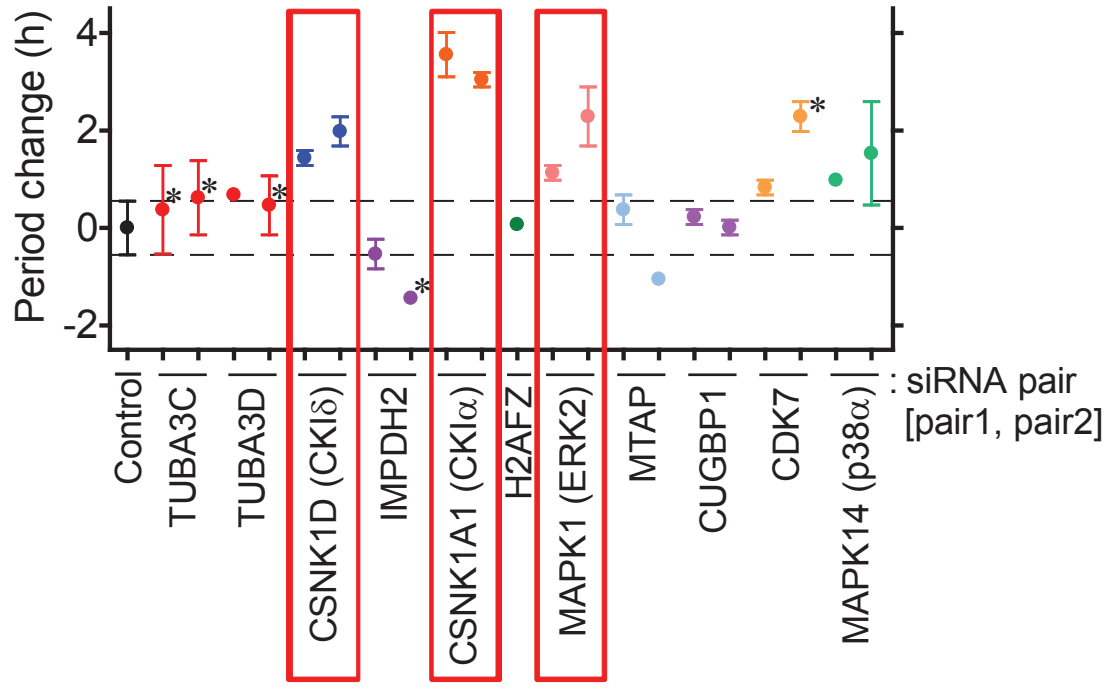

Supplement: Figure S4 — Effects of siRNAs against longdaysin-binding proteins on the circadian period. Data are extracted from the primary screen of our genome-wide RNAi study [33]. Luminescence rhythms of Bmal1-dLuc U2OS cells were monitored after transient transfection with siRNAs. Four independent siRNAs were tested per gene as two siRNA pools (#1 and #2), each containing two independent siRNAs. Data are the mean with variation (n = 2). Asterisk indicates low amplitude rhythm, which sometimes causes wrong period estimation because of poor curve fitting. Pairs #1 and #2 for H2AFV and pair #1 for H2AFZ have highly possible off-target genes, and the result is not shown. (0.23 MB PDF) [file pbio.1000559.s004.pdf]

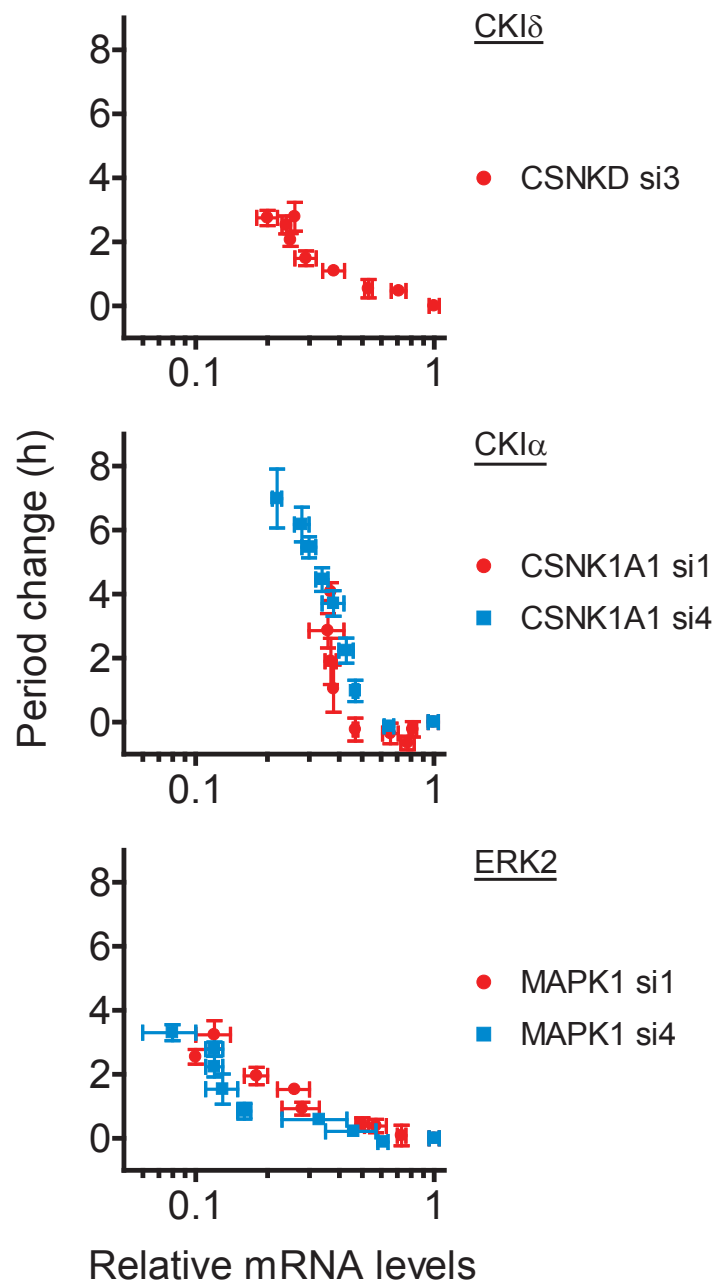

Supplement: Figure S5 — Comparison of the effect of individual siRNA on the circadian period and the target gene expression. Target gene knockdown effect from Figure 3Cii (x-axis in log scale; the mean with variation, n = 2) was plotted against period effect from Figure 3Ci (y-axis; the mean with SEM, n = 5–6) for each siRNA. (0.24 MB PDF) [file pbio.1000559.s005.pdf]

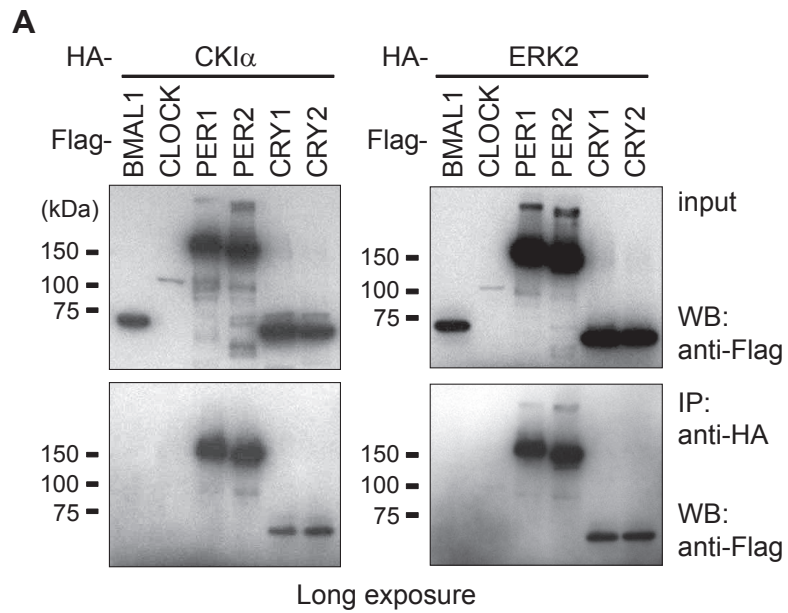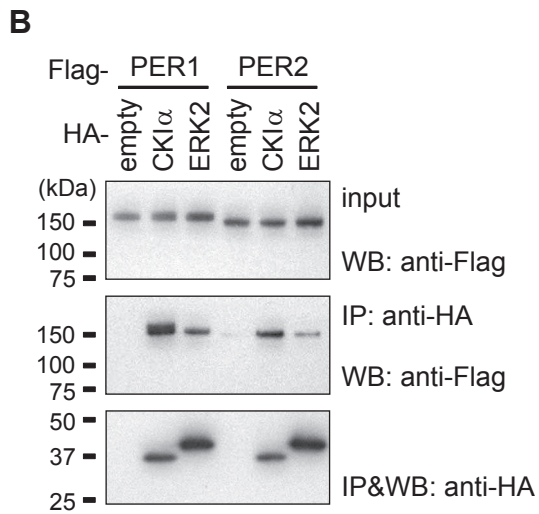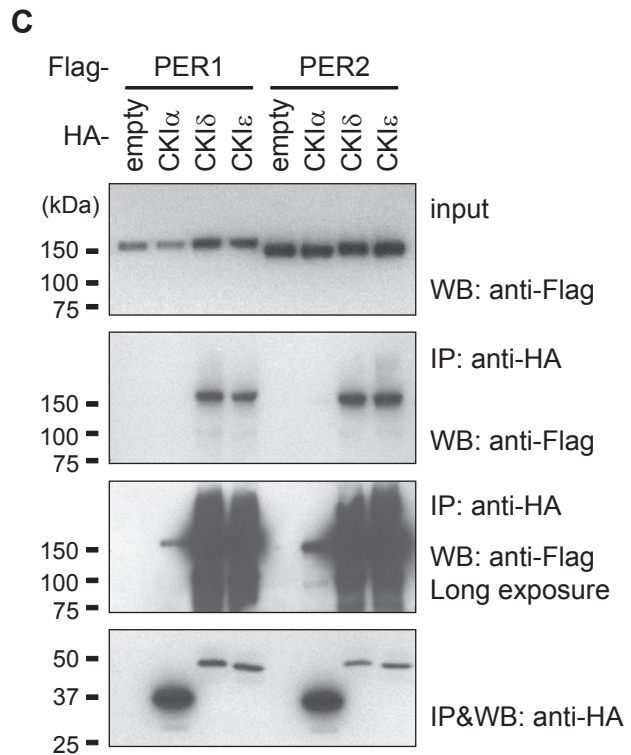

Supplement: Figure S6 — Interaction of CKIα and ERK2 with the clock proteins. (A) Long exposure images of Figure 4A. (B) Interaction of CKIα and ERK2 with PER1/2. (C) Interaction of CKIα, CKIδ, and CKIε with PER1/2. HA-tagged kinases were co-expressed with Flag-tagged clock proteins in HEK293T cells and subjected to immunoprecipitation assay with anti-HA antibody. (1.35 MB PDF) [file pbio.1000559.s006.pdf]

**A**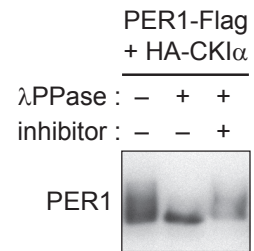**B**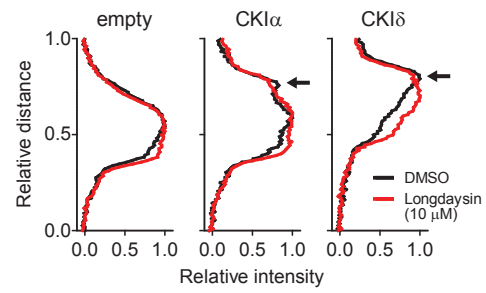**C**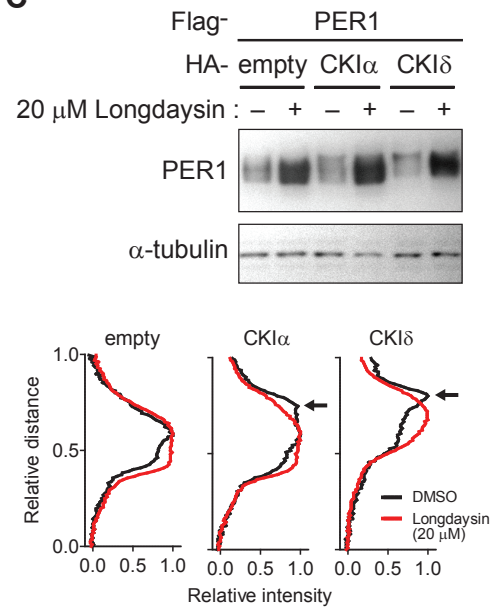

Supplement: Figure S7 — Effect of longdaysin on CKIα- and CKIδ-dependent phosphorylation of PER1. (A) HEK293T cell extract expressing PER1 and CKIα was treated with λ protein phosphatase in the absence or presence of phosphatase inhibitor and analyzed by Western blot. (B and C) PER1 was co-expressed with CKIα or CKIδ in HEK293T cells. The cells were treated with longdaysin (10 µM in B and 20 µM in C) or DMSO for 24 h and analyzed by Western blot. Intensity profile of each PER1 band is shown in (B) and bottom panels of (C) by setting the peak value as 1. Western blot image for (B) is shown in Figure 4C. Arrows indicate (sub)peaks of PER1, which appeared depending on CKIα or CKIδ and shifted by longdaysin treatment. (0.56 MB PDF) [file pbio.1000559.s007.pdf]

**A**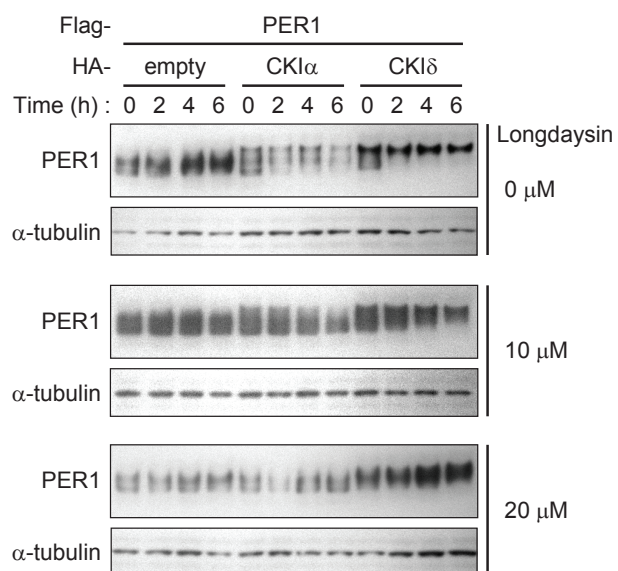**B**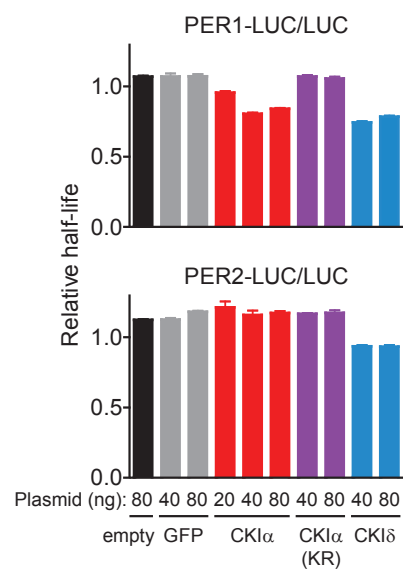**C**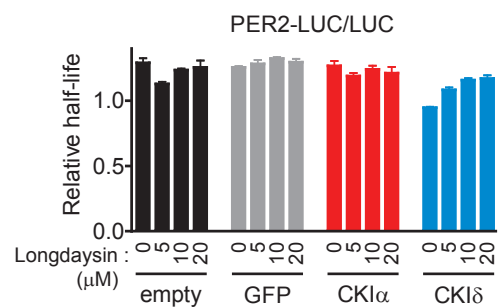

Supplement: Figure S8 — Effect of longdaysin on CKIα- and CKIδ-dependent regulation of PER1 and PER2 stability. (A) Flag-tagged PER1 was co-expressed with CKIα or CKIδ in HEK293T cells. The cells were treated with 0, 10, or 20 µM longdaysin for 24 h and then treated with cycloheximide from time 0. The cells were collected 0, 2, 4, or 6 h later and analyzed by Western blot. Note that co-expression of CKIα or CKIδ accelerated degradation of PER1 compared with empty vector control (upper panels, 0 µM longdaysin). The effects of CKIα and CKIδ were partially inhibited by 10 µM longdaysin (middle panels) and strongly inhibited by 20 µM longdaysin (lower panels). (B) PER1-LUC, PER2-LUC, or LUC was co-expressed with various amounts of GFP, CKIα, CKIα (KR), or CKIδ in HEK293T cells. The cells were treated with cycloheximide from time 0, and luminescence was recorded. The relative half-life of PER1-LUC (upper panel) or PER2-LUC (lower panel) against LUC is indicated. Data are the mean with SEM (n = 4). Note that CKIα did not affect PER2-LUC stability, while its effect on PER1-LUC was saturated at 80 ng condition. (C) PER2-LUC or LUC was co-expressed with GFP, CKIα, or CKIδ in HEK293T cells. The cells were treated with 0 to 20 µM longdaysin for 24 h and then treated with cycloheximide for luminescence recording. The relative half-life of PER2-LUC against LUC is indicated. Data are the mean with SEM (n = 4). (0.96 MB PDF) [file pbio.1000559.s008.pdf]

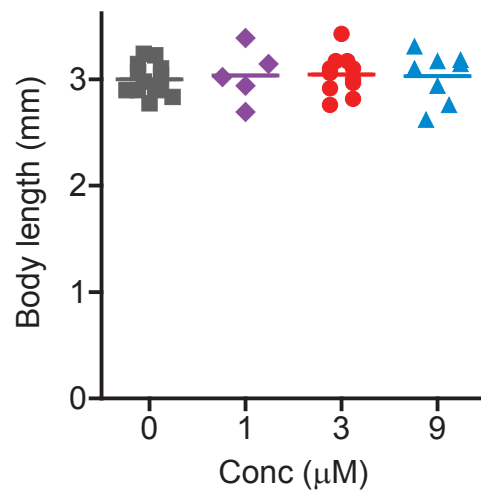

Supplement: Figure S9 — Effect of longdaysin on body length of zebrafish. Zebrafish were treated with longdaysin for luminescence recording (Figure 5). Body length of zebrafish was measured after the 1 wk treatment and plotted against compound concentration (n = 13, 5, 11, and 8 for 0, 1, 3, and 9 µM longdaysin, respectively). (0.21 MB PDF) [file pbio.1000559.s009.pdf]

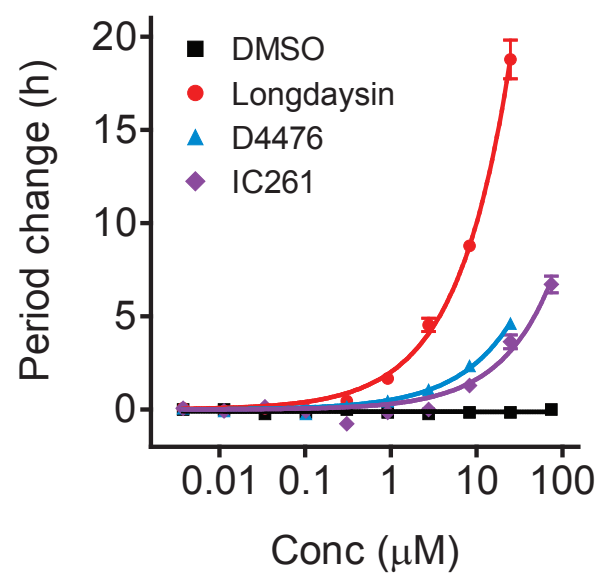

Supplement: Figure S10 — Effects of CKI inhibitors D4476 and IC261 on the circadian period. Luminescence rhythms of Bmal1-dLuc U2OS cells were monitored in the presence of various concentrations of compounds. Data are the mean with SEM (n = 4). Longdaysin and D4476 showed cytotoxicity at 71 µM. (0.23 MB PDF) [file pbio.1000559.s010.pdf]
