# Supplementary material for: High-Throughput Chemical Screen Identifies a Novel Potent Modulator of Cellular Circadian Rhythms and Reveals CKIα as a Clock Regulatory Kinase
Source: PLoS Biol. 2010 Dec 14;8(12):e1000559. doi: 10.1371/journal.pbio.1000559 (PMC3001897; doi:10.1371/journal.pbio.1000559)
Supplement: Table S1 — siRNA sequences. (0.22 MB PDF) [file pbio.1000559.s011.pdf]

Name	Sense Sequence	Antisense Sequence
CSNK1D si3	CUCACAGGCCGACAAGAUACCUCTC	GAGAGGUAUCUUGUCGGCCUGUGAGGU
CSNK1A1 si1	GCCCGAUAUGCUAGCAUCAAUGCAC	GUGCAUUGAUGCUAGCAUAUCGGGCAG
CSNK1A1 si4	AGAAUUUGCGAUGUACUUAAACUAT	AUAGUUUAAGUACAUCGCAAAUUCUGC
MAPK1 si1	CCAUCGCCGAAGCACCAUUCAAGTT	AACUUGAAUGGUGCUUCGGCGAUGGGC
MAPK1 si4	GCACCAACCAUCGAGCAAAUGAAAG	CUUUCAUUUGCUCGAUGGUUGGUGCUC
