# Supplementary material for: High-Throughput Chemical Screen Identifies a Novel Potent Modulator of Cellular Circadian Rhythms and Reveals CKIα as a Clock Regulatory Kinase
Source: PLoS Biol. 2010 Dec 14;8(12):e1000559. doi: 10.1371/journal.pbio.1000559 (PMC3001897; doi:10.1371/journal.pbio.1000559)
Supplement: Table S2 — qPCR primer sequences. (0.24 MB PDF) [file pbio.1000559.s012.pdf]

Name	Forward sequence	Reverse sequence
CSNK1D	CCCATCGAAGTGTTGTGTAAAGG	GCCGCAGGTACGAGTAGTCA
CSNK1E	AAGACGGTGCTGCTCTTGG	GAGGAAGTTGTCGGGCTTG
CSNK1A1	ATGGTTGGCTTCTTGTCTGC	TCACTTTAGATGCTGTTATTTCTAGCAC
CSNK1A1L	CTTCTTGTCTGTAAGCCAGC	TCTTATGTCTTCACAGGTAAGC
MAPK1	CTAGATTCCAGCCAGGATACAG	AGAAGAACACCGATGTCTGAG
MAPK3	GTACTATGACCCGACGGATG	GAGATGTCTGTCTGGGCTAG
ACTB	CATGTACGTTGCTATCCAGGC	CTCCTTAATGTCACGCACGAT
GAPDH	TGCACCACCAACTGCTTAGC	ACAGTCTTCTGGGTGGCAGTG
