# Supplementary material for: High-Throughput Chemical Screen Identifies a Novel Potent Modulator of Cellular Circadian Rhythms and Reveals CKIα as a Clock Regulatory Kinase
Source: PLoS Biol. 2010 Dec 14;8(12):e1000559. doi: 10.1371/journal.pbio.1000559 (PMC3001897; doi:10.1371/journal.pbio.1000559)
Supplement: Text S1 — Supporting methods. Synthesis of compound 1, Longdaysin, compound 2, and compound 3. (0.49 MB DOC) [file pbio.1000559.s013.doc]

**Text S1**

**High-Throughput Chemical Screen Identifies a Novel Potent Modulator of Cellular Circadian Rhythms and Reveals CKIα as a Clock Regulatory Kinase**

**Tsuyoshi Hirota, Jae Wook Lee, Warren G. Lewis, Eric E. Zhang, Ghislain Breton, Xianzhong Liu, Michael Garcia, Eric C. Peters, Jean-Pierre Etchegaray, David Traver, Peter G. Schultz, and Steve A. Kay**

**Supporting Methods**

**Synthesis of compound 1, Longdaysin, compound 2, and compound 3**

General

All chemicals and solvents were obtained from commercial suppliers (Acros and Aldrich) and used without further purification. Unless otherwise indicated, all reactions were performed under argon gas. Anhydrous solvents were obtained via passage through an activated alumina column. ^1^H NMR spectra were recorded on a Bruker 500 MHz spectrometer. Chemical shifts are reported relative to internal CDCl_3_ (Me_4_Si, δ 0.0), CD_3_OD (Me_4_Si, δ 0.0) and DMSO-*d*_6_ (δ 2.50 for ^1^H).

All compounds were identified by LC-MS from Agilent Technology, using a C_18_ column (20 × 4.0 mm), with 3.5 minutes elution using a gradient solution of CH_3_CN/H_2_O (containing 0.05% trifluoroacetic acid), with UV detector and an electrospray ionization source.

**Scheme I. Synthesis of compound 1 and logdaysin (5).** (a) Isopropanol (2.0 eq), DEAD (1.2 eq), PhP_3_ (2.0 eq), 0^o^C, overnight. (b) 3-trifluoromethylbenzylamine (1.52 eq), DIEA (1.2 eq), n-BuOH, 60^o^C, overnight.

Synthesis of 6-chloro-9-isopropyl-9H-purine (**4**)

To a solution of chloropurine (2 g, 13.0 mmol) in THF (160 mL) was added isopropanol (1.6 g, 26.0 mmol, 2.0 eq), DEAD (2.71 g, 15.6 mmol, 1.2 eq) and Ph_3_P (6.8 g, 26.0 mmol, 2.0 eq) at 0^o^C. The reaction mixture was then stirred at room temperature overnight. After the reaction was complete, the mixture was extracted with methylene chloride (5 x 50 mL). The combined organic extracts were dried with MgSO_4_ and filtered. The solution was concentrated *in vacuo*. The reaction mixture was purified by silica gel column chromatography by elution with CH_2_Cl_2_/n-hexane/MeOH=1:1:0.05 to afford **4** as a white solid (1.8 g, 82%).

^1^H-NMR (500 MHz, CDCl_3_): δ=1.7 ppm (6H, d, J=7.0 Hz), 4.9 ppm (1H pentet, J=7.0 Hz), 8.19 ppm (1H, s), 8.76 ppm (1H, s).

LC-MS (ESI) calcd C_8_H_9_ClN_4_ (M+1)=197.1 found: 197.1

Synthesis of N-benzyl-9-isopropyl-9H-purin-6-amine (**1**)

To a solution of **4** (50 mg, 0.25 mmol) in n-BuOH (2 mL) was added DIEA (48 μL, 0.3 mmol, 1.2 eq) and benzylamine (42 μL, 0.38 mmol, 1.52 eq). After heating at 60^o^C overnight, solvent was evaporated. The reaction mixture was purified by RP-HPLC [CH_3_CN/H_2_O=10% to 70% (containing 0.05% trifluoroacetic acid), *R*_t_ = 3.2 min] to afford **1** as a white solid (34.2 mg, 51.2%).

^1^H-NMR (500 MHz, DMSO-*d_6_*): δ=1.58 ppm (6H, d, J=6.5 Hz) 4.85 ppm (3H, m), 5.35 ppm (1H, m) 7.33 ppm (1H, t, J=7) 7.38 ppm (2H, t, J=7) 7.43 ppm (2H, d, J=7) 8.47 ppm (1H, s) 8.69 ppm (1H, s).

LC-MS (ESI) calcd for C_15_H_17_N_5_ (M+1)=268.2 found: 268.2

Synthesis of 9-isopropyl-N-(3-(trifluoromethyl)benzyl)-9H-purin-6-amine (longdaysin) (**5**)

To a solution of **4** (240 mg, 1.22 mmol) in n-BuOH (4 mL) was added DIEA (234 μL, 1.46 mmol, 1.2 eg) and 3-trifluoromethyl benzylamine (262 μL, 1.85 mmol, 1.52 eg). After heating at 60^o^C overnight, solvent was evaporated. The reaction mixture was purified by RP-HPLC [CH_3_CN/H_2_O=10% to 70% (containing 0.05% trifluoroacetic acid), *R*_t_ = 2.9 min] to afford **5** as a white solid (241 mg, 55.6%).

^1^H-NMR (500MHz, DMSO-*d*_6_): δ=1.58 ppm (6H, d, J=7), 4.83 ppm (3H, m), 7.60 ppm (1H, t, J=7.5), 7.65 ppm (1H, d, J=7.5), 7.72 ppm (1H, d, J=7.5), 7.78 ppm (1H, s), 8.3 ppm (1H, s), 8.4 ppm (1H, s).

LC-MS (ESI) calcd for C_16_H_16_F_3_N_5_ (M+1)=336.2, found: 336.2

**Scheme II. Synthesis of compounds 2 and 3.** (a) Isopropanol alcohol (2.0 eq), DEAD (1.2 eq), PhP_3_ (2.0 eq), 0^o^C, overnight. (b) 3-trifluoromethylbenzylamine (1.1 eq), DIEA (1.2 eq), n-BuOH 60^o^C, overnight. (c) Boc-aminohexyl amine (3.0 eq), DIEA (2.0 eq), n-BuOH, 120^o^C, overnight. (d) 10% TFA/MC, room temperature, 1 hour. (e) Affi-gel 10 agarose bead (0.01 mmol/mL) triethylamine (10.0 eq), room temperature, overnight, 2-aminoethanol (10.0 eq), room temperature, overnight. (f) DIEA (2.0 eq), acetic anhydride (1.0 eq), room temperature, overnight.

Synthesis of 2, 6-dichloro-9-isopropyl-9H-purine (**6**)

To a solution of 2, 6-dichloropurine (800 mg, 4.23 mmol) in THF (20 mL) was added isopropanol (508 mg, 8.46 mmol, 2.0 eq), DEAD (787 μL, 5.0 mmol, 1.2 eq) and Ph_3_P (2.2 g, 8.46 mmol, 2.0 eq) at 0^o^C. The reaction mixture was then stirred at room temperature overnight. After the reaction was complete, the mixture was extracted with methylene chloride (3 x 50 mL). The combined organic extracts were dried with MgSO_4_ and filtered. The solution was concentrated *in vacuo*. The reaction mixture was purified by column chromatography (by elution with n-hexane 100% to ethyl acetate/n-hexane=1:1) to afford **6** as a white solid (830.8 mg, 85.4%).

^1^H NMR (500 MHz, CDCl_3_): δ=1.6 ppm (6H, d, J=6.5), 5.0 ppm (1H, m), 8.2 ppm (1H, s).

LC-MS (ESI) calcd for C_8_H_8_Cl_2_N_4_ (M+1)=231.0 found: 231.0

Synthesis of 9-isopropyl-N-(3-(trifluoromethyl)benzyl)-9H-purin-6-amine (**7**)

To a solution of **6** (860 mg, 3.74 mmol) in n-BuOH (4 mL) was added DIEA (724 μL, 4.48 mmol, 1.2 eq) and 3-trifluoromethyl benzylamine (589 μL, 4.11 mmol, 1.1 eq). After heating at 60^o^C overnight, solvent was evaporated. The reaction mixture was purified by column chromatography (by elution with n-hexane 100% to ethyl acetate/n-hexane/MeOH=2:1:0.01) to afford **7** as a white solid (856 mg, 62%).

^1^H NMR (500 MHz, CD_3_OD): δ=1.6 ppm (6H, d, J=7), 4.8 ppm (1H, m), 4.94 ppm (2H, s), 7.6 ppm (1H, t, J=7.5), 7.65 ppm (1H, m), 7.7 ppm (1H, m), 7.78 ppm (1H, S), 8.2 ppm (1H, s).

LC-MS (ESI) calcd for C_16_H_15_ClF_3_N_5_ (M+1)=370.1 found: 370.1

Synthesis of N2-(6-aminohexyl)-9-isopropyl-N6-(3-(trifluoromethyl)benzyl)-9H-purine-2,6-diamine (**8**)

To a solution of **7** (160 mg, 0.43 mmol) in n-BuOH (4 mL) was added DIEA (139 μL, 0.86 mmol, 2.0 eq) and 1-Boc-aminohexyl-6-amine (260 mg, 1.2 mmol, 3.0 eq). After heating at 120^o^C for 10 hrs, the mixture was extracted with methylene chloride (5 x 20 mL). The combined organic extracts were dried with MgSO_4_ and filtered. The solution was concentrated *in vacuo*. Crude compound was filtered through silicagel. Without further purification, the reaction mixture was treated with 10% trifluoroacetic acid/CH_2_Cl_2_ (10 mL). The reaction mixture was stirred at room temperature for 1 hour. After solvent was evaporated, the reaction mixture was purified by RP-HPLC [CH_3_CN/H_2_O=10% to 50% (containing 0.05% trifluoroacetic acid), *R*_t_ = 2.1 min] to afford **8** as a white solid (98 mg, 50.7%).

^1^H NMR (500 MHz, CD_3_OD): δ=1.38 ppm (4H, m), 1.6 ppm (6H, d, J=7), 1.61 ppm (2H, m), 1.69 ppm (2H, m), 2.9 ppm (2H, m), 3.4 ppm (2H, m), 4.8 ppm (2H, s), 7.45 ppm (1H, t, J=7.5), 7.5 ppm (1H, m), 7.68 ppm (1H, m), 7.72 ppm (1H, S), 8.1 ppm (1H, s).

LC-MS (ESI) calcd for C_22_H_30_F_3_N_7_ (M+1)=449.3, found: 449.3

Synthesis of compound **3**

To a solution of **8** (4.49 mg, 0.01 mmol) in DMSO was added triethylamine (13.5 μL, 0.1 mmol, 10.0 eq) and agarose bead [Affi-gel 10 Gel (Bio-Rad 153-6046), activated by *N*-hydroxysuccinimide, (0.01 mmol/mL), (3.3 mL, 3.3 eq)]. After shaking at room temperature overnight, the reaction was analyzed by LC-MS. After 12 hours, LC-MS indicated that all of the starting material had been consumed, and the reaction mixture was treated with 2-ethanolamine (6.1 mg, 0.1 mmol, 10.0 eq) and allowed to stir at room temperature overnight. The reaction mixture was filtered through frit. The agarose beads were washed with DMSO (2mL, 3X) and PBS (2mL, 3X). The agarose beads were stored at 4^o^C in 0.05% NaN_3_ in PBS solution.

Synthesis of N-(6-(9-isopropyl-6-(3-(trifluoromethyl)benzylamino)-9H-purin-2-ylamino)hexyl)acetamide (**2**)

To a solution of **8** (49.4 mg, 0.11 mmol) in THF (2 mL) was added DIEA (35.5 μL, 0.22 mmol, 2.0 eq) and acetic anhydride (11.22 mg, 0.11 mmol, 1.0 eq) at 0^o^C. After stirring at room temperature overnight, solvent was evaporated. The reaction mixture was purified by RP-HPLC [CH_3_CN/H_2_O=10% to 70% (containing 0.05% trifluoroacetic acid), *R*_t_ = 2.3 min] to give **2** as yellow oil (25.3 mg, 46.3%).

^1^H NMR (500 MHz, CD_3_OD): δ=1.42 ppm (4H, m), 1.55 ppm (2H, m), 1.66 ppm (6H, d, J=7), 1.97 ppm (3H, s), 3.2 ppm (2H, m), 3.5 ppm (2H, m), 4.75 ppm (1H, m), 4.9 ppm (2H, m), 7.65 ppm (2H, m), 7.76 ppm (2H, m), 8.2 ppm (1H, s).

LC-MS (ESI) calcd for C_24_H_32_F_3_N_7_O (M+1)=492.3, found: 492.3
